# Fluorescence Sensing Using DNA Aptamers in Cancer Research and Clinical Diagnostics

**DOI:** 10.3390/cancers9120174

**Published:** 2017-12-20

**Authors:** Domenica Musumeci, Chiara Platella, Claudia Riccardi, Federica Moccia, Daniela Montesarchio

**Affiliations:** 1 Department of Chemical Sciences, University of Napoli Federico II, Via Cintia 21, 80126 Napoli, Italy; domenica.musumeci@unina.it (D.M.); chiara.platella@unina.it (C.P.); claudia.riccardi@unina.it (C.R.); mocciafederica@gmail.com (F.M.); 2 CNR, Istituto di Biostrutture e Bioimmagini, Via Mezzocannone 16, 80134 Napoli, Italy; 3 CNR, Istituto per l’Endocrinologia e l’Oncologia “Gaetano Salvatore,” Via Pansini 5, 80131 Napoli, Italy

**Keywords:** DNA aptamers, fluorescence sensing, fluorescently-labelled aptamers, label-free aptamers, cancer biomarkers, tumour diagnosis

## Abstract

Among the various advantages of aptamers over antibodies, remarkable is their ability to tolerate a large number of chemical modifications within their backbone or at the termini without losing significant activity. Indeed, aptamers can be easily equipped with a wide variety of reporter groups or coupled to different carriers, nanoparticles, or other biomolecules, thus producing valuable molecular recognition tools effective for diagnostic and therapeutic purposes. This review reports an updated overview on fluorescent DNA aptamers, designed to recognize significant cancer biomarkers both in soluble or membrane-bound form. In many examples, the aptamer secondary structure switches induced by target recognition are suitably translated in a detectable fluorescent signal using either fluorescently-labelled or label-free aptamers. The fluorescence emission changes, producing an enhancement (“signal-on”) or a quenching (“signal-off”) effect, directly reflect the extent of the binding, thereby allowing for quantitative determination of the target in bioanalytical assays. Furthermore, several aptamers conjugated to fluorescent probes proved to be effective for applications in tumour diagnosis and intraoperative surgery, producing tumour-type specific, non-invasive in vivo imaging tools for cancer pre- and post-treatment assessment.

## 1. Introduction

### 1.1. Nucleic Acid Aptamers and Their Selection Process

**Figure 1:**
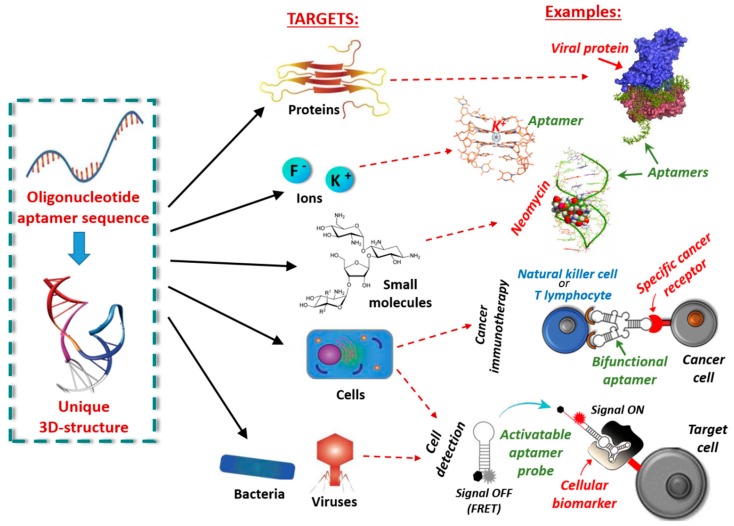
Schematic representation of possible targets of oligonucleotide aptamers and corresponding examples.

Nucleic acid aptamers are short DNA or RNA molecules (oligonucleotides, ONs), or related analogues, able to bind with high affinity and specificity a wide range of targets including metal ions, organic dyes, nucleotides, amino acids, peptides, enzymes, proteins, whole cells and even entire organisms, such as viruses or bacteria, thanks to their unique three-dimensional folding (Figure 1) [1]. They can form various secondary structures (e.g., stems, loops, bulges, pseudoknots, G-quadruplexes and kissing hairpins) [2], which in turn can give rise to unique three-dimensional architectures able to specifically recognize their targets by exploiting a variety of interactions—such as hydrophobic and electrostatic interactions, hydrogen bonding, van der Waals forces and π-π stacking—as well as shape complementarity.

**Figure 2:**
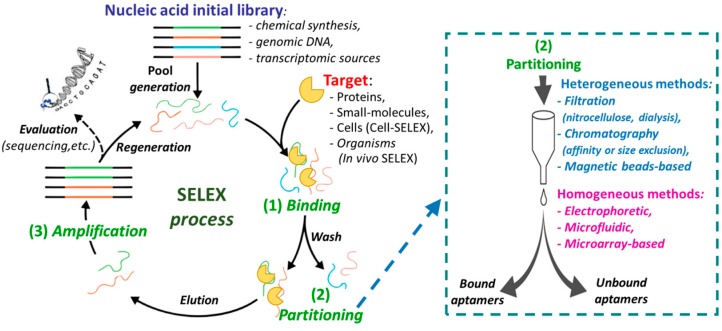
Schematic representation of the Systematic Evolution of Ligands by Exponential Enrichment (SELEX) process steps; on the right, available partitioning methods are listed.

Aptamers are generally identified through a methodology known as Systematic Evolution of Ligands by Exponential Enrichment (SELEX), an in vitro iterative process that allows the selection, from libraries of 10^15^–10^16^ different oligonucleotide sequences, of high affinity ligands for the chosen chemical or biological target [3]. The aptamers obtained by SELEX exhibit dissociation constants (K_d_) towards the targets in the micromolar to femtomolar range. The SELEX methodology essentially consists of four steps: (i) the binding procedure, in which an ON library, containing a 20/60 nucleotide random sequence flanked by fixed primer regions at the 5′- and 3′- ends, is incubated with the target molecule under defined experimental conditions (e.g., specific library/target ratio, buffer, ionic strength, pH, temperature, or incubation time); (ii) the separation of the target-bound aptamers from unbound ONs (partitioning); (iii) the elution of the bound sequences from the target (recovery); (iv) the amplification of the enriched pool of selected aptamers before the successive selection round (Figure 2). The ON libraries to evolve aptamers can be obtained using chemical synthesis, genomic DNA [4] or transcriptomic sources [5] and can contain natural or modified nucleotides [6,7,8,9]. The use of modified nucleotides can increase the chemical and enzymatic stability of aptamers, as well as improve their binding affinity and specificity. The modified monomers can be inserted after the SELEX procedure (post-SELEX modifications) or, more efficiently, included in the initial ON library, thanks to the availability of engineered mutant polymerases able to recognize also nucleotide analogues [10,11,12].

After incubation of the ON library with the selected target, the separation of bound from unbound sequences can be realized by both heterogeneous methods (filtration, affinity chromatography, or magnetic bead-based separation), or by homogeneous partition techniques, such as kinetic capillary electrophoresis methodologies (Figure 2, right) [13,14]. Then the bound aptamers are eluted from the target and amplified by PCR (DNA SELEX) or reverse transcription followed by PCR (RNA SELEX) to give an enriched pool of selected ONs (Figure 2), even if, in some cases, amplification is not strictly necessary [15].

After several selection rounds, cloning into a plasmid and sequencing are performed to identify high affinity aptamers. A significant improvement in the SELEX procedure has been obtained with the application of next-generation sequencing (NGS) combined with bioinformatic analysis of the growing aptamer populations, enabling the identification of structural motifs that might be critical for the aptamer–target interaction [16,17]. The identified aptamers are then synthesized and studied to determine their target affinity and specificity of binding vs. other possible targets.

Structural characterization of the aptamer and of its complex with the target is crucial for optimizing the aptamer recognition abilities. Computational analysis, biophysical studies (NMR, X-ray crystallography) and, more importantly, biochemical assays allow identifying the molecular details of the aptamer–target interaction [18,19,20]. Information on the binding pocket and the residues critical for target contacts are essential for aptamer optimization.

**Figure 3:**
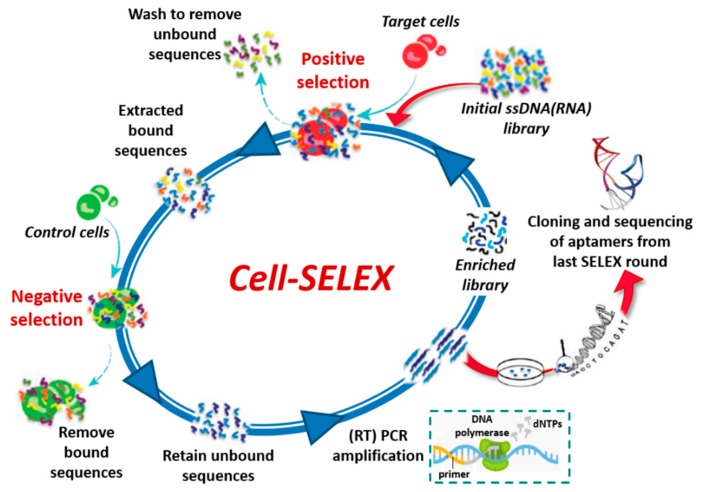
Schematic representation of cell-SELEX process.

The SELEX process can be also applied to select aptamers targeting whole living cells (cell-SELEX, Figure 3) [21,22] providing an effective approach for, e.g., (i) identification of new biomarkers as disease signals in diagnosis and therapy [23,24], (ii) visualization and capture of cancer cells, (iii) targeting of cell surface receptors to increase the effectiveness of the immune system in cancer immunotherapy [25] and (iv) recognition of virus-infected cells [26]. Cell-SELEX does not necessitate of purified proteins, since the aptamers are selected against membrane proteins in their native conformation. In addition, it allows the identification of aptamers against biomarkers differentially expressed between different cell lines without prior knowledge of the targets. Generally, a counter-selection (negative selection) with control cells is inserted in the cell-SELEX procedure before or immediately after the positive selection with the target cells (Figure 3).

### 1.2. Therapeutic and Diagnostic Applications of Nucleic Acid Aptamers

Nucleic acid aptamers are considered an effective alternative to protein antibodies [27] in therapeutics (as drugs or drug-delivery systems) [28,29,30,31,32,33] and bio-analysis (as diagnostic and sensing tools) [34,35,36,37,38,39] due to their smaller size, lower immunogenicity, remarkable stability over a wide range of temperatures and pH (≈4–9) and easy control of their folding processes, typically reversible on restoring the native conditions. In addition, once selected, ON-based aptamers are easily obtained by chemical synthesis. This allows also to introduce ad hoc tailored chemical modifications aimed at increasing the aptamer chemical and biological stability, also bringing the production costs well below those of antibodies [40]. Furthermore, aptamers can be easily equipped with a wide variety of reporter groups and coupled to different probes, nanoparticles, or other biomolecules.

In contrast to this extraordinary potential, to date there is only one aptamer-based drug approved by the US Food and Drug Administration: Macugen (pegaptanib sodium), selected against the vascular endothelial growth factor (VEGF) and used for the treatment of neovascular age-related macular degeneration (AMD) in humans [41]. Several aptamers are currently in advanced clinical trials but there is no current indication that a few of them may enter in clinical practice in short times.

Despite the limited commercial success with aptamer-based therapeutics, the recent progress in aptamer selection and formulation should encourage scientists to continue in the search for therapeutic aptamers. Indeed, proof-of-concept studies in aptamer technology fully validate their promising functionality and vast therapeutic potential. 

In therapeutics, aptamers can be exploited as: (i) antagonists to block the interaction of disease-associated targets, such as protein-protein, protein-nucleic acid, receptor-ligand interactions [28]; (ii) agonists to activate the function of target receptors [42,43,44]; or (iii) carriers to deliver other therapeutic agents to the target cells or tissue [45]. All the aptamers currently in advanced clinical trials fall into the first category.

Several research groups have developed ON-based aptamers against therapeutically relevant targets associated with several human diseases [46], including ocular [47] or bleeding [48] disorders, cancer, cardiovascular, autoimmune, degenerative neurological [49] and infectious diseases [50,51].

In addition to their use as stand-alone therapeutic agents, aptamers may be exploited as escorts for the delivery of another drug. Various cell-type-specific aptamers have been applied in association with therapeutic agents—for example, small interfering RNAs (siRNAs), microRNAs (miRNAs), antimiRs, therapeutic aptamers, chemotherapeutics or toxins—or delivery carriers (organic or inorganic nanocarriers) for targeted delivery in a cell-type-specific manner. By taking advantage of the high affinity and specificity of aptamers, drugs can be targeted to the desired cells or tissues, thereby improving their local concentration and therapeutic efficacy [45].

In diagnostics, nucleic acid aptamers have emerged in the design of novel biosensors (“aptasensors”) for the detection of infective agents, antigens (bacteria), toxins, disease biomarkers (cancer), as well as specific metal ions. In the sensing process, aptamers often recognize their targets by a mechanism in which they undergo considerable structural rearrangements [52], which can be exploited for transducing the binding event into a measurable signal. Multiple sensing strategies have been developed thus far, combined with various transducers, such as quartz crystal microbalance (QCM), surface plasmon resonance (SPR), fluorescence, colorimetry, electrophoresis, electrochemistry, electrochemiluminescence (ECL), field-effect transistor, etc. [53,54,55,56,57].

Among them, optical analysis has been extensively developed because of high sensitivity, quick response and simple operation mode and numerous optical aptasensors have been designed for the detection of small molecules, proteins and metal ions [58,59,60,61].

For many sensor platforms, aptamers have to be immobilized for signal production and in these cases detection can be achieved by different sensing modes, among which electrochemical and optical are the most used ones. Soluble aptamers can also be employed for homogeneous assays, or assays that only use a liquid phase, mainly based on measurements of absorbance, fluorescence or chemiluminescence [62]. Both in heterogeneous or homogeneous aptamer-based sensing systems, aptamers can be labelled with fluorescent molecules, enzymes, quantum dots (QDs) and nanoparticles (NPs), or used in label-free form. Aptamer labelling is usually time-consuming, cost effective and affects the binding affinity and selectivity to the target. However, this is a still unbeatable strategy for revealing aptamer structural changes upon target recognition.

### 1.3. Aptamer-Sensing Systems Based on Fluorescence

Fluorescence is one of the most commonly used optical techniques and has been widely applied to produce aptasensors for its high sensitivity, efficiency, operation simplicity and low costs. While the fluorescence signal of fluorescently-labelled antibodies generally remains the same whether or not the antibody is bound to the target, ligand-induced transitions of the aptamer secondary structures can be conveniently monitored using covalently-attached luminescent labels or non-covalent luminescent probes. The aptamer-based fluorescent biosensors can be broadly divided into fluorescently-labelled aptamers and label-free aptamers. Within each category, there are “signal-on” and “signal-off” sensor-based strategies, typically employing fluorescence resonance energy transfer (FRET). The signal change, either an increase (i.e., the “signal-on” mode) or a decrease (i.e., the “signal-off” mode), reflects the extent of the binding process, thereby allowing for quantitative measurements of the target concentration.

**Figure 4:**
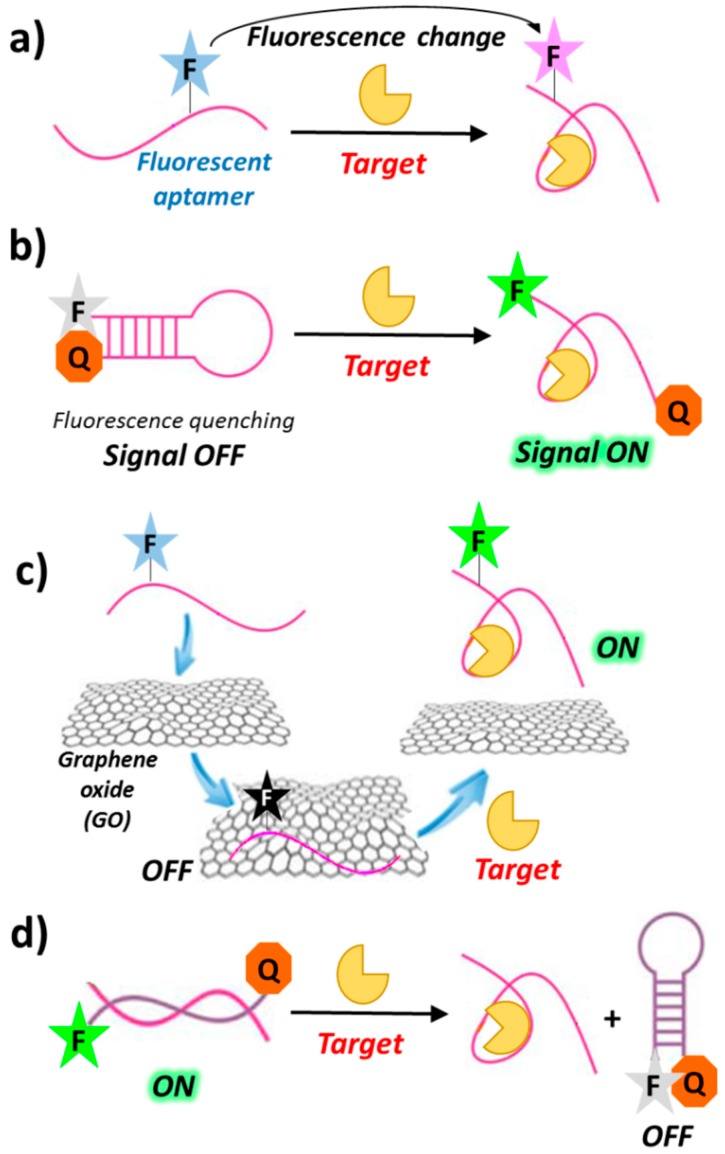
Schematic representation of general strategies exploited for the design of fluorescently-labelled aptamer biosensors, based on fluorescence changes (**a**), “signal-on” mode in solution (**b**) or assisted by nanomaterials (**c**) and “signal-off” mode induced by target recognition (**d**). F = fluorophore; Q = quencher; Target = protein, small molecule, ion, etc.

The simplest design of a fluorescent aptasensor consists of a single fluorophore covalently linked to the aptamer—within its backbone or at the termini—undergoing significant structural reorganization following target binding. In this system, target-aptamer interaction is evidenced by either an increase or a decrease in fluorescence intensity resulting from the change in the electronic environment surrounding the fluorophore upon ligand binding (Figure 4a) [63,64].

In a typical “signal-on” biosensor exploiting fluorescently-labelled aptamers, a fluorophore is conjugated at one end and a quencher at the other end of the aptamer (molecular or aptamer beacons). In the absence of its target, the aptamer is folded into a hairpin conformation, thereby positioning the fluorophore close to the quencher (quenching by FRET). Upon ligand binding, base-pairing in the hairpin is interrupted and the aptamer converts to its open conformation, in which the fluorophore and the quencher do not interact (Figure 4b). The resulting recovery of the fluorescence signal can be used for detection and quantitative measurement of the target concentration [65]. Other “signal-on” strategies are those exploiting graphene surfaces. Indeed, graphene oxide (GO) exhibits intriguing electronic properties which make it an excellent energy acceptor and hence an effective fluorescence quencher (Figure 4c) [66,67,68].

Although sensors based on the “signal-off” mode are usually less sensitive than those based on the “signal-on” mode [69,70], they can sometimes lead to a better detection of the targets with low-affinity aptamers [71,72]. Examples of this sensing design generally require a fluorescence donor and a quencher conjugated at the termini of the oligonucleotide complementary to the chosen aptamer. In the duplex aptamer-complementary oligonucleotide, the two probes on the complementary strand are far apart, thus showing high fluorescence. Upon target binding, conformational changes of the aptamer drive the donor and the quencher close to each other on the complementary strand, leading to fluorescence quenching (Figure 4d).

**Figure 5:**
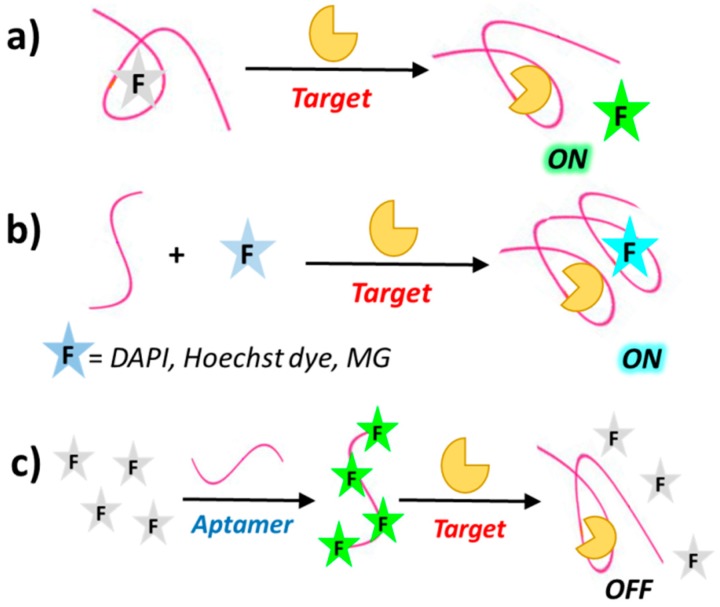
Schematic representation of different strategies exploited in the development of label-free aptamer biosensors, based on “signal-on” mode, after fluorophore displacement (**a**) or fluorophore binding (**b**) and “signal-off” mode after fluorophore displacement (**c**), induced by target recognition. DAPI = 4′,6-diamidino-2-phenylindol; MG = malachite green.

Despite the many interesting applications of biosensors based on fluorescently-labelled aptamers, covalent labelling of aptamers with fluorophores can be time-consuming and expensive. In addition, the conjugated fluorophore may interfere with target binding of the aptamer. To overcome these drawbacks, several strategies have been developed to design biosensors based on label-free aptamers. One common strategy for “signal-on” aptamer-based biosensors involves the use of label-free aptamers which, upon target binding, can displace fluorophores that are either already quenched (by, e.g., gold nanoparticles) or poor fluorescent (Figure 5a). Similarly to the above mentioned “signal-on” biosensors using fluorescently-labelled aptamers, label-free aptamers can also activate fluorophores by either deactivation or removal of the quencher [73]. Furthermore, some fluorescent dyes such as 4′,6-diamidino-2-phenylindol (DAPI), Hoechst 33,258 [74] and malachite green (MG) [75] are very weakly fluorescent when free in solution but exhibit enhanced fluorescence after aptamer binding as a result of target-induced conformational switch (Figure 5b).

The “signal off” sensing mode of label-free aptamers can be obtained through, for example, fluorophore displacement upon aptamer binding, as in the case of the dye OliGreen (OG), which exhibits more than 1000-fold enhanced fluorescence upon binding to single stranded DNA and is displaced as a consequence of the ON conformational changes induced by target recognition (Figure 5c) [76].

The proper sensing design is conceived considering the following information: (i) the unbound aptamer conformation; (ii) the behaviour of the aptamer in the presence and absence of the reporter groups, both in covalently-linked or free form; (iii) the conformation of the aptamer in the target-bound form, as well as some molecular details of the aptamer–target interaction. In addition, the aptamer can be also easily engineered to add some modular elements (stem, loop, etc.) suitable for dye recognition.

### 1.4. Aim of This Review

We here present an updated overview on fluorescent aptamers and aptamer-based fluorescence strategies applied in the recognition of relevant cancer biomarkers, essentially proteins/receptors, both in soluble or cancer cell membrane-bound forms. We analyse examples in which the protein-mediated transition of the aptamer folding can be conveniently transduced in a fluorescent signal using both fluorescent labels covalently attached to the aptamers or non-covalently-linked fluorescent probes (label-free aptamers) and employing different sensing strategies (“signal-on,” “signal-off” mode, etc.) for potential theranostic applications.

A number of important targets are discussed, from thrombin—which, even if marginally involved in cancer-related pathologies, represents the most studied protein for aptamer recognition, typically exploited as a proof-of-concept in sensing strategies validation—to other important cancer biomarkers including platelet-derived growth factor (PDGF), vascular endothelial growth factor (VEGF), angiogenin and mucin. In addition, several aptamer-based fluorescence approaches developed to allow tumour cells detection for diagnosis and intraoperative surgery are also described as valuable examples of in vivo/in vitro imaging applications.

## 2. Aptamer-Based Fluorescent Systems for the Specific Recognition of Cancer-Related Targets

Several aptamers have been selected against targets implicated in tumorigenesis, identified either against cancer-related purified proteins, or against complex matrices (e.g., serum, plasma, blood), enabling in the latter case the discovery of new cancer biomarkers. In parallel, the efficient targeting ability of aptamers to cancer cells and tissues can also provide a promising strategy to deliver imaging agents and drugs for tumour visualization and therapy. Therefore, it is possible to generate aptamer-based probes with the ability to recognize cancer cells or cancer-related soluble proteins.

In this frame, a number of researches are focused on detection and/or treatment of cancer-related targets using fluorescent aptasensors.

### 2.1. Thrombin

For its physiological role in thrombosis, hemostasis and other coagulation-related reactions [77,78,79,80,81,82], thrombin is a crucial protein; altered thrombin concentration levels in the blood are known to be associated with a variety of diseases, including Alzheimer and cancer [83,84,85].

The amount of thrombin in blood is generally low in non-pathological conditions but it increases after proteolysis of prothrombin and is secreted in the blood reaching concentrations from pM to µM levels in case of diseases [86,87]. For this reason, the correct quantification of thrombin in blood is extremely important and bioanalytical assays with sufficiently low detection limits are needed.

The most studied aptamers able to recognize the human α-thrombin are the 15-mer and the 29-mer thrombin binding aptamers (TBAs). Both of them are DNA oligonucleotides, binding the protein at two different anion binding sites, i.e., the exosite I and exosite II, respectively. The 15-mer DNA oligonucleotide 5′-GGT TGG TGT GGT TGG-3′, named TBA_15_ or simply TBA, first described by Bock and co-workers in 1992 [88], can form a stable antiparallel G-quadruplex (G4) structure with a chair-like conformation [89]. This oligonucleotide interacts with the exosite I, i.e., the fibrinogen-recognition site, with a dissociation constant of ca. 100 nM (for a recent review on TBA features see for example reference [90]). On the other hand, the 29-mer DNA oligonucleotide TBA_29_ 5′-AGT CCG TGG TAG GGC AGG TTG GGG TGA CT-3′, folding into a modular G4/duplex structure, binds to the heparin-binding site (exosite II) of thrombin with a K_d_ of 0.5 nM [91].

The great enthusiasm behind the use of thrombin as model for the development of protein detection protocols (more than one hundred analytical techniques focused on thrombin detection are reported by Deng and co-workers [92]), especially those based on fluorescence sensing systems, is largely due to the peculiar features of these TBAs. Indeed, both are short oligonucleotides, relatively easy to synthesize and—if necessary—modify with fluorescence probes or reporter groups such as quencher tags. In addition, the capability to form a G4 structure induced by thrombin recognition is a key feature in the development of conformation-switched systems with a “turn-on” or “turn-off” signal according to the structure adopted by the aptamer in solution. Finally, the 15-mer and the 29-mer oligonucleotides, recognizing the two different thrombin binding sites without mutual interference, can be used in tandem in detection protocols based on sandwich-type systems. Thus, the TBAs currently represent the best studied models to validate a sensing strategy. Valuable examples of thrombin detection using fluorescent thrombin binding aptamers have been widely summarized in previous works [92,93,94]. We here describe a selection of recent sensing strategies using fluorescent TBAs.

**Figure 6:**
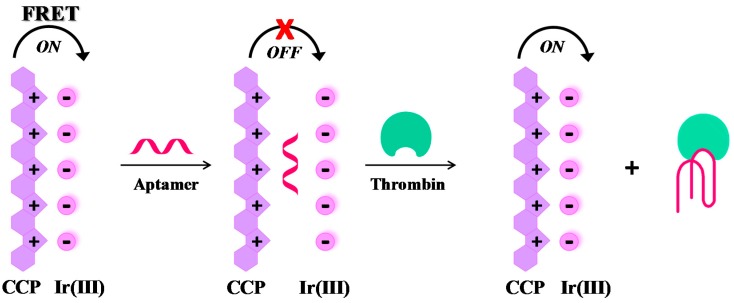
Schematic representation of a label-free, “signal-on” sensing strategy for the selective detection of thrombin, as described in reference [97].

Label-free, “signal-on” sensing strategies. Many effective label-free, “signal-on” sensing strategies have been reported in the literature, as those described by Yan et al. [95] and Zhang et al. [96]. More recently, a novel, label-free thrombin detection assay has been developed based on the fluorescence resonance energy transfer (FRET) from a cationic conjugated polymer (CCP), acting as the energy donor, to an Ir(III) complex, acting as the acceptor (Figure 6). In this system, the energy donor and the acceptor are kept in close proximity by π-π stacking and electrostatic interactions, leading to fluorescence enhancement by FRET. When TBA_29_ is present, the proposed order between CCP and Ir(III) complex is disturbed and the FRET between CCP and Ir(III) complex is inconspicuous (quenching effect). However, in the presence of thrombin the folding of the aptamer results in fluorescence restoring, thus allowing the quantitative detection of thrombin (“signal-on” mode). This assay permits the detection of thrombin up to 0.05 pM concentration, with a high discrimination ability towards other proteins [97].

**Figure 7:**
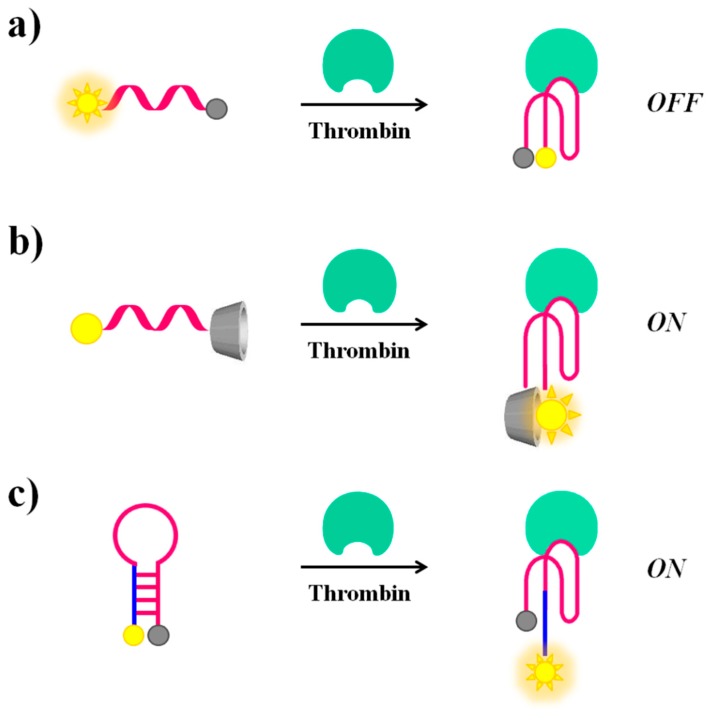
Schematic representation of molecular beacons undergoing conformational changes upon thrombin recognition: switch from a random coil to a G4 structure, causing a “turn-off” (**a**) or a “turn-on” (**b**) of the fluorescence signal and from a hairpin to a G4 structure (**c**), with a “turn on” of the fluorescence emission.

Molecular beacons. A valuable example of fluorescent molecular beacons has been designed by Li et al., who conjugated through short linkers 6-FAM (6-carboxyfluorescein) and dabcyl (4-4′-dimethylaminophenylazo benzoic acid), acting respectively as the fluorophore and the quencher probes, at the 5′- and 3′-ends of the 17-mer TBA-related sequence (5′-T GGT TGG TGT GGT TGG T-3′) [98]. The oligonucleotide exists in dynamic equilibrium between a non-structured random coil and a compact intramolecular antiparallel G-quadruplex structure. In the presence of thrombin, this equilibrium is shifted towards the G-quadruplex, leading the fluorophore and the quencher in close proximity, thus resulting in a “turn-off” of the fluorescence signal (Figure 7a). This molecular aptamer beacon recognizes its target with a detection limit of 112 pM concentration.

In contrast, the thrombin recognition can also produce a “turn-on” effect on the fluorescence signal of suitably modified TBA, as reported by De Tito et al. [99]. In this work, a fluorescent TBA_15_ conjugated with the environmentally sensitive dansyl [5-(dimethylamino)naphthalene-1-sulfonyl)] probe and a β-cyclodextrin residue, respectively at the 3′- and 5′-ends, has been efficiently prepared (Figure 7b). When the oligonucleotide is in the random coil form, the dansyl group, exposed to the bulk water, shows only basal fluorescence [100]. On the contrary, when TBA_15_ folds into an antiparallel G-quadruplex structure, as for example upon thrombin recognition, a dramatic fluorescence enhancement is observed. This occurs since, in the folded state, the dansyl group is encapsulated in the hydrophobic cavity of the β-cyclodextrin ring resulting in a net fluorescence enhancement [99]. As a further development of this work, Riccardi and co-workers have described a tris-conjugated TBA_15_ (tris-mTBA), equipped with a dansyl, a β-cyclodextrin and a biotin tag at the ends. This novel design has allowed the incorporation of TBA_15_ onto streptavidin-coated NPs, leading to a remarkable increase of its anticoagulant properties. The developed systems have provided the basis for suitable aptamer-based devices for theranostic applications, allowing simultaneously both fluorescence-based detection and modulation of the thrombin activity [101].

Notably, in addition to the sensing approaches based on conformational switch random coil-G-quadruplex structure, also thrombin-induced changes starting from a hairpin structure are possible if the aptamer is properly engineered. In this context, Hamaguchi et al. have described a TBA_15_ elongated at the 5′-end with few nucleotides complementary to the 3′-end and therefore able to adopt a stem-loop structure or hairpin [102]. In addition, the aptamer is equipped with a fluorescent/quencher pair, i.e., a fluorescein and a dabcyl moiety at the 5′- and 3′-end, respectively. In the absence of thrombin, the close proximity between the two reporter groups in the hairpin structure determines fluorescence quenching. After thrombin recognition, the stem-loop structure is destabilized in favour of interactions with the protein. Under these conditions, the fluorescent dye and the quencher are distant, thus allowing a “turn-on” of the fluorescence signal, indicative of the binding with the target molecule (Figure 7c).

**Figure 8:**
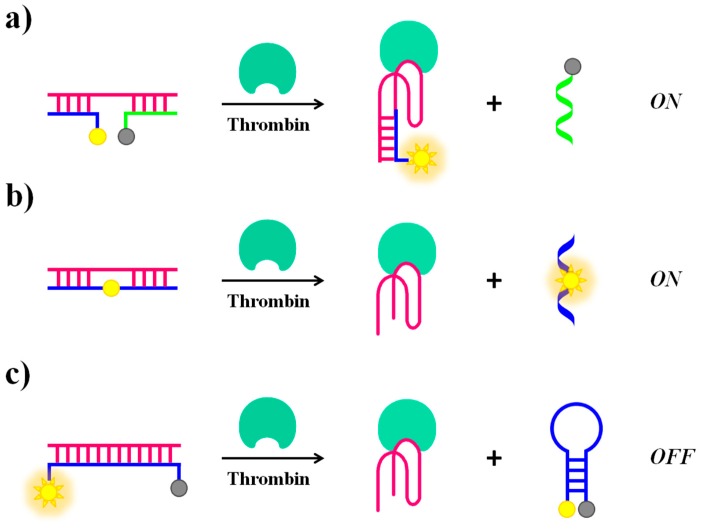
Schematic representation of molecular beacons strategies based on “structure switch signalling aptamer” from a duplex structure to a G4 induced by addition of thrombin causing “turn on” (**a**,**b**) or “turn-off” (**c**) of the fluorescence signal.

Alternative approaches for “structure switch signalling aptamers” are reported by Nutiu and Li [103]. Their strategy for designing aptamer-based fluorescent reporters involves structural switches from DNA/DNA duplex to DNA/target complex. In this study, the aptamer beacon consists of a tripartite duplex structure including a 5′-fluorescein-labeled oligomer (FDNA), a 3′-dabcyl-labeled oligomer (QDNA) and a longer oligonucleotide sequence comprising Stem-1 and Stem-2, complementary to FDNA and QDNA, respectively. Stem-2 also contains the TBA_15_ sequence in a partial overhang (Figure 8a). In the absence of the target protein, the aptamer naturally binds to FDNA and QDNA, bringing the fluorophore and the quencher in close proximity and thus completely inhibiting the fluorescence signal. The presence of thrombin triggers the formation of the aptamer-target complex, causing the release of QDNA and fully restoring the fluorescence emission.

Li et al. proposed a general strategy for a competitive molecular beacon that does not require modifications on the original aptamer but only of the DNA competitor, demonstrating the efficiency both in a “turn-on” (Figure 8b) and a “turn-off” (Figure 8c) detection [70]. In the signal-on detection scheme (Figure 8b), a fluorescent nucleotide analogue, such as 2-aminopurine and pyrrolo-dC, is incorporated in the competitor oligonucleotide sequence. The fluorescence quantum yield of these nucleotide analogues is strictly dependent on the stacking interactions with their neighbour bases. Initially, fluorescence quenching in the double-stranded competitor/aptamer complex occurs. The fluorescence signal is then recovered when the competitor/aptamer duplex is dissociated upon addition of the target. In the signal-off detection scheme (Figure 8c), the fluorophore and quencher are located each at one end of the competitor oligonucleotide, complementary to the aptamer sequence, allowing the initial detection of high fluorescence signal relative to the competitor/aptamer duplex. When displaced by the target, the competitor changes from an “open” conformation to a “closed” hairpin conformation that brings the fluorophore and the quencher in close proximity, thus leading to a fluorescence decrease.

A further development of the molecular beacon approach is based on nanomaterial-assisted assays which involve the use of quantum dots, gold nanoparticles, magnetic NPs, graphene or single-walled carbon nanotubes (SWCTs) acting themselves as a fluorophore or a quencher probe without the need of synthetic modifications on the aptamer (or of the competitor) sequence. Suitable nanomaterials have been successfully exploited for both single strand- and duplex-based strategies.

**Figure 9:**
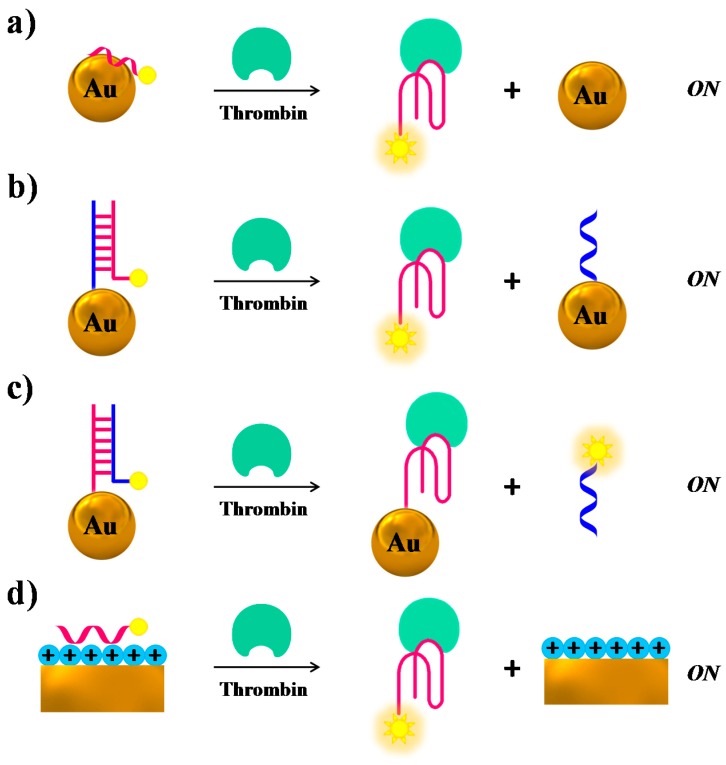
Schematic representation of thrombin detection by fluorescence strategies based on nanomaterial-assisted assays where gold nanoparticles (**a**–**c**) or gold-based nanostructures (**d**) are used as fluorescence quenchers, as described in references [104,105]. In all cases, the addition of thrombin “turns on” the fluorescence signal.

Several research groups have used gold nanoparticles (AuNPs) or nanostructures as fluorescence quenchers, combined with modified versions of TBA_15_ as a probe: generally, upon target binding a “turn-on” of the fluorescence after spatial separation of the fluorophore from the Au surfaces is obtained. Fluorescent TBA_15_ derivatives have been attached to AuNPs through either aspecific adsorption [104] (Figure 9a), or duplex formation with TBA complementary sequences covalently linked to the NPs [104] (Figure 9b,c); in another case they have been linked to chitosan-coated Au nanostructures through electrostatic attractions [105] (Figure 9d), always resulting in quenching effects. Upon thrombin binding, the aptamers form a G-quadruplex structure, so dissociating from the gold surfaces with subsequent fluorescence recovery. In the case of AuNPs the detection limits are strictly connected to the nanoparticle size, reaching 0.14 and 0.46 nM limits in 5- and 10-nm size, respectively. On the contrary, the chitosan/Au-based sensor showed a detection limit of 10 pM. 

Another example of structural switch causing fluorescence enhancement, similar to the case of Figure 9b, is provided by a system in which the TBA-complementary sequence is labelled with the fluorophore, whereas the aptamer is covalently linked to the AuNPs surface. Thus, the duplex formation on the NPs promotes fluorescence quenching, while in the presence of thrombin the target-TBA complex formation, triggered by the NPs, displaces the fluorescently-labelled complementary strand, which produces a net fluorescence emission (Figure 9c) [104].

**Figure 10:**
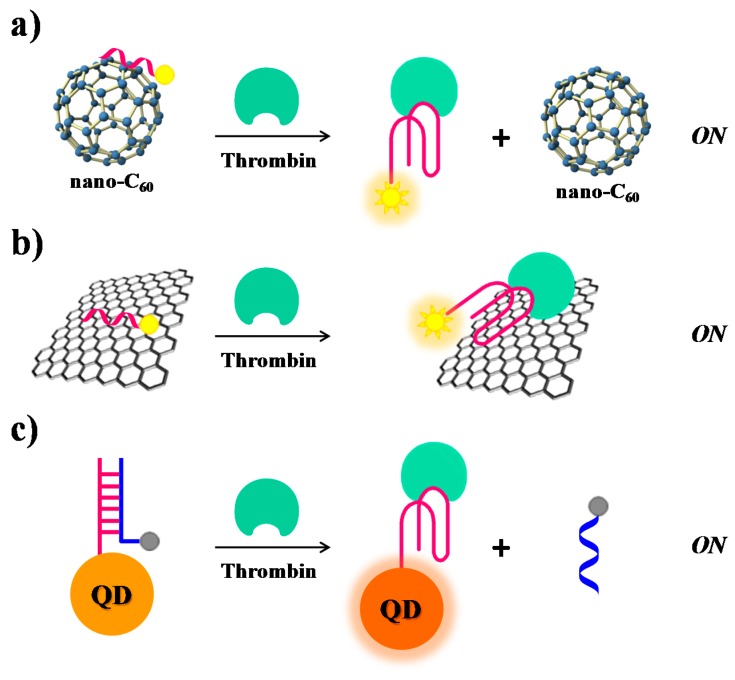
Schematic representation of thrombin detection strategies based on nanomaterial-assisted fluorescence assays employing (**a**) nano-C_60_ [107] (**b**) graphene [68] and (**c**) quantum dots (QDs) [111]. In all these cases, the thrombin recognition “turns on” the fluorescence signal.

Furthermore, also SWCTs, graphene and magnetic NPs are able to adsorb single-stranded DNA and can efficiently induce fluorescence quenching. Thus, in various similar designs, FAM-labelled-aptamers have been adsorbed on SWCTs [106], nano-C_60_ (Figure 10a) [107], or graphene (Figure 10b) [68], whereas a Cy3-labeled-TBA_15_ has been linked to magnetic nanoparticles (MNPs) [108], resulting in all cases in fluorescence quenching effects. In the presence of the target, the formation of the G4-thrombin complex causes an increase of the distance between the fluorescent group on the aptamer and the nanomaterial, leading to “turn on” the fluorescence signal. In these approaches, the detection limits for thrombin are estimated to be 1.8 nM for SWCTs, 1 nM for nano-C_60_, 31.3 pM in the case of the graphene FRET aptasensor and 0.5 nM in the case of MNPs (Table 1).

The intrinsic fluorescence of quantum dots (QDs) has been exploited by Levy and co-workers [111]. They have covalently attached TBA_15_ on QDs and allowed it forming a duplex structure with the complementary sequence, previously labelled with a quencher group, thus obtaining quenched QDs (Figure 10c). After addition of thrombin, the TBA conformational changes induced the displacement of the quencher-labelled complementary strand, thus restoring the original fluorescence signal of QDs (signal-on).

Sandwich-type recognition strategy. Another interesting approach in the development of efficient thrombin binding assays is based on the thrombin recognition by two aptamers and formation of a DNA assembly.

Heyduk et al. [112] have used two fluorescently-labelled aptamers to comprise a FRET pair: TBA_29_, labelled with fluorescein as the fluorescence donor [5′-fluorescein AGA TGCG—(Spacer18)_5_ AGT CCG TGG TAG GGC AGG TTG GGG TGA CT-3′] and TBA_15_ with dabcyl as the fluorescence acceptor [5′-GGT TGG TGT GGT TGG—(Spacer18)_5_—CGC ATC T-3′-dabcyl]. In both oligonucleotides a long linker (containing 5 Spacer18 units) has been introduced to produce a better affinity for thrombin [112]. The co-association of the aptamers with the protein leads the two “signalling” oligonucleotides into proximity, producing large fluorescence changes, which allow effective thrombin quantification even at 1 nM concentration (Table 1). 

Catalytic beacons. Catalytic beacons make use of specific DNA sequences able to catalyse various reactions and for this reason are known as DNAzymes.

**Figure 11:**
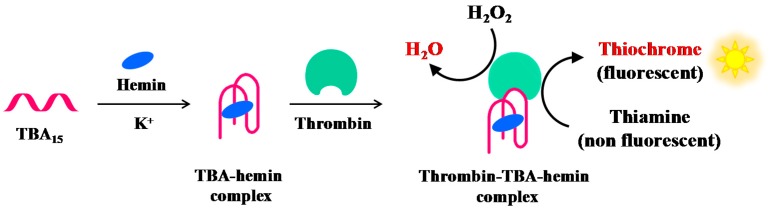
Schematic representation of the fluorescence thrombin detection based on the DNAzyme formation between the hemin-TBA complex and thrombin, as described in reference [109].

Zhang et al. have reported a label-free and sensitive fluorescence method for detection of thrombin using a G-quadruplex-based DNAzyme (Figure 11). TBA_15_ is able to bind hemin forming a complex with moderate peroxidase-like activity, thus generating a G-quadruplex-based DNAzyme whose activity is significantly enhanced in the presence of thrombin. The G4-based DNAzyme effectively catalyses the H_2_O_2_-mediated oxidation of thiamine (a non-fluorescent substrate), giving rise to fluorescence emission caused by thiochrome formation. The combination of TBA_15_-hemin-thrombin system with the H_2_O_2_–thiamine fluorescent reaction allows detection of thrombin up to 1 pM [109].

Enzyme-assisted assays. Many protein binding assays take advantage of standard methods combined with enzymatic amplification of target molecules and enhancement of detection signals in order to improve their sensitivity. In particular, the cyclic enzymatic signal amplification (CESA) method has been developed for highly sensitive detection of nucleotides, DNA and proteins [146]. This method utilizes enzymatic reactions or other signal amplification mechanisms to transduce target binding events into measurable signals.

However, specific recognition sites are often required for selected enzymes and, in the case of CESA for oligonucleotides, nicking endonucleases (NEase), a special family of restriction endonucleases cutting only one strand of a double-stranded DNA, are the most frequently used enzymes.

**Figure 12:**
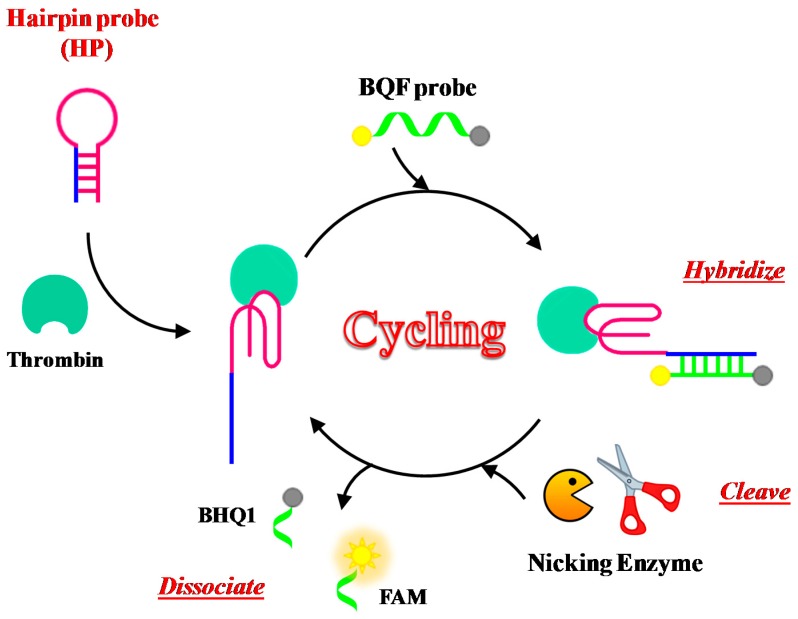
Schematic representation of the fluorescence thrombin detection based on NEase-assisted cyclic enzymatic signal amplification (CESA), as described in reference [113]. The cleavage of the BQF (BHQ-quenching fluorescence) probe by nicking endonuclease regenerates the fluorescence signal. FAM = 6-carboxyfluorescein; BHQ1 = Black Hole Quencher 1.

Xue et al. have developed a new assay for thrombin detection based on a hairpin probe and nicking enzyme-assisted signal amplification strategy, named NEase-assisted CESA [113]. The hairpin probe contains the TBA_15_ sequence for target recognition as well as a long stem designed to be complementary to the BHQ-quenching fluorescence (BQF) probe (Figure 12). The BQF probe is a short ON including the recognition sequences and cleavage site for the nicking endonuclease, labelled with the fluorescent dye 6-carboxyfluorescein (6-FAM) and its quencher Black Hole Quencher 1 (BHQ1) at the 5′- and 3′-ends, respectively. 

Binding to the target leads to conformational changes of the hairpin probe, allowing its hybridization with the BQF probe. The fluorescence signal is thus amplified through continuous enzyme cleavage of the BQF probe to remove the BHQ moiety. This method can detect thrombin with a detection limit of 100 pM.

Combining the sandwich-type method described above and the enzymatic strategy, Xue et al. achieved efficient thrombin detection also using aptamer–protein–aptamer conjugates and nicking enzyme assisted fluorescence signal amplification assay (NEFSA), improving the detection limit of thrombin to 40 pM [147].

**Figure 13:**
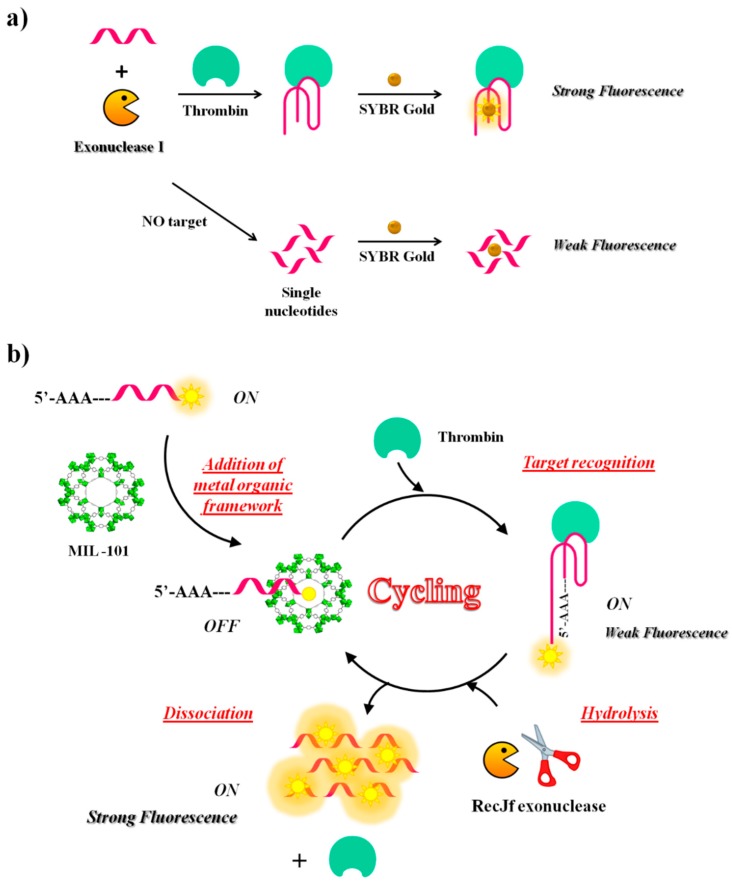
Schematic representation of two different strategies based on exonucleases digestion activity: (**a**) structure-switching aptamer after thrombin binding, protected from Exo I digestion and subsequently detected with SYBR Gold, as described in reference [110]; (**b**) fluorescence amplification strategy based on aptamer-triggered directional hydrolysis upon target recognition, as described in reference [148].

In addition to endonucleases, also exonucleases were used in the CESA method, as reported by Zheng and co-workers [110]. They have developed a sensing strategy employing structure-switching aptamers (SSAs), exonuclease I (Exo I) and SYBR Gold—one of the most commonly used dyes for nucleic acid staining—to detect a broad range of targets including thrombin (Figure 13a). The oligonucleotide used in this study as antithrombin aptamer has the following sequence: 5′-AGT CCG TGG TAG GGC AGG TTG GGG TGA CT-3′. Once the structure-switching aptamer binds the target, it folds into a G-quadruplex, which is more resistant to nuclease digestion than the single strand in unfolded conformations. The amount of aptamer left after nuclease reaction is proportional to the concentration of the targets and is in turn proportional to the fluorescence intensities from SYBR Gold that can only stain nucleic acids but not their digested fragments. This aptamer successfully detected thrombin in the human serum at 680 nM concentration (Table 1).

Another fluorescence amplification strategy for selective and sensitive detection of thrombin is based on aptamer-triggered directional hydrolysis and use of metal organic framework (MOF) of type MIL-101 (Cr_3_F(H_2_O)_2_O[(O_2_C)-C_6_H_4_-(CO_2_)]_3_ · nH_2_O), a crystalline porous nanomaterial prepared through coordination interactions between metal ions and organic ligands. The method is implemented by the initial mixing of MIL-101, well known for its strong binding to negatively charged single-stranded DNA through π-π stacking and electrostatic interactions and a fluorescein-labelled DNA probe, realizing a strong fluorescence quenching (Figure 13b). After addition of exonuclease of type RecJf and the target, the aptamer is released from MIL-101 upon target recognition; exonuclease hydrolysis, directionally-assisted by the insertion of three adenines on the DNA probe, occurs, thus producing a fluorescence signal amplification. Good selectivity is accomplished in this strategy due to the use of MIL-101-protected aptamers, while high sensitivity results from exonuclease-assisted target-recycling signal amplification. The method has been fully validated using two model analytes, i.e., thrombin, a fairly large protein and oxytetracycline (OTC), a small antibiotic molecule. The detection limit is 15 pM for thrombin and 4.2 nM for OTC. The strategy has been successfully applied to the analysis of thrombin in real samples [148].

The detection strategies described above for thrombin can be extended to other important cancer-related targets; some of them have already been explored in this sense, as described below.

### 2.2. PDGF

The human platelet-derived growth factor (PDGF) is a protein found in human serum and platelets that plays important roles in embryogenesis [149] and in regulation of cell growth, division and migration [150,151,152]. PDGF monomeric forms, each containing a different polypeptide chain, i.e., PDGF-A, -B, -C and -D, are inactive [149,153] but become biologically active after dimerization through formation of disulphide bonds. Four homodimers (PDGF-AA, -BB, -CC and -DD) and one heterodimer (PDGF-AB) are currently known. PDGF isoforms produce their cellular effects by specific binding to PDGF membrane receptors [154,155]. Mutations and overexpression of PDGF proteins and receptors are involved in proliferation of cancer cells, angiogenesis and development of tumour-associated fibroblasts in solid tumours [156,157,158,159]. Therefore, PDGF dimeric proteins attract great interest as prognostic and treatment markers in cancers [160].

In 1996, Green et al. identified by SELEX an oligonucleotide able to recognize PDGF-BB with high affinity and selectivity compared to the other isoforms [161]. This PDGF-BB aptamer folds into a three-way helix junction with a three-nucleotide loop at the branch point [161]. The high affinity of the aptamer-protein interaction (K_d_ ≈ 10^−10^ M) has been exploited in all the sensing strategies described below [114,115,117,118,120,121,123,162], guaranteeing higher specificity towards PDGF-BB than the other isoforms and proteins present in the analysed samples.

**Figure 14:**
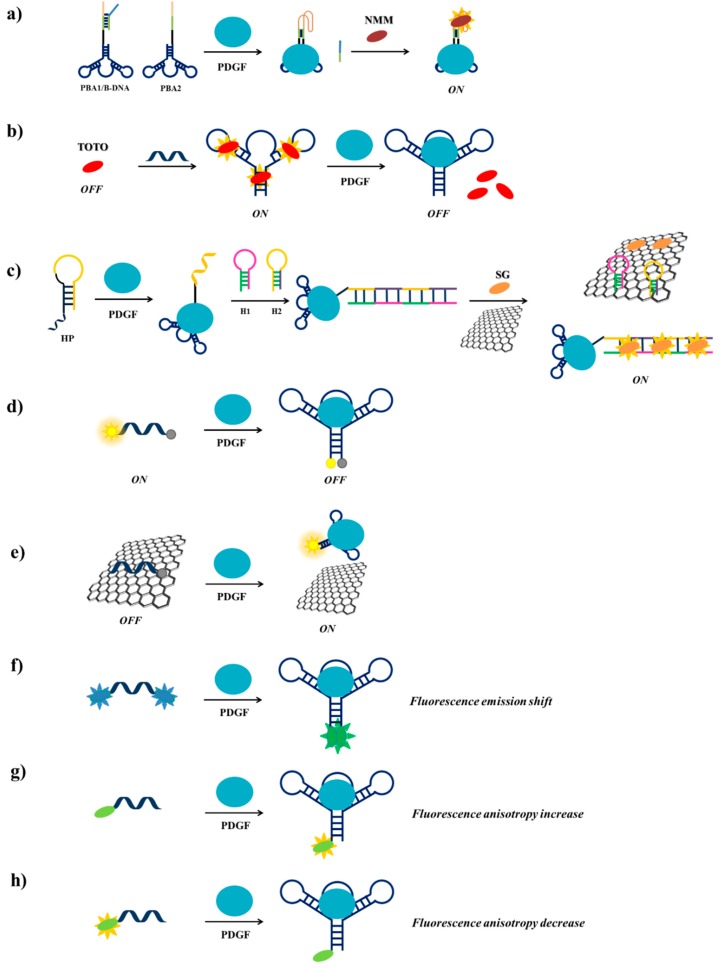
Schematic representation of PDGF detection strategies using label-free (**a–c**) or fluorescently-labelled (**d–h**) aptamers. PBA1 = PDGF-BB binding aptamer 1; PBA2 = PDGF-BB binding aptamer 2; NMM = *N*-methylmesoporphyrin IX; TOTO = 1,1′-(4,4,8,8-tetramethyl-4,8-diazaundecamethylene)-bis-4-[3-methyl-2,3-dihydro(benzo-1,3-thiazole)-2-methylidene] quinolinium tetraiodide; HP = helper DNA probe; H1 = hairpin probe 1; H2 = hairpin probe 2; SG = SYBR Green I.

Among the strategies for PDGF detection based on PDGF-BB-targeting aptamers, Wei et al. [114] have described a label-free fluorescent detection based on the aptamer proximity binding assay strategy [163]. Three different oligonucleotides are required in this approach: (i) PBA1 and PBA2 (PDGF-BB binding aptamer 1 and 2), each constituted by a G4-forming sequence, a complementary sequence, a T_25_ spacer, introduced to avoid steric hindrance effects in the successive assay and the PDGF-BB aptamer sequence and (ii) B-DNA, a single strand DNA able to hybridize to the complementary sequences and to part of the G4-forming sequences of PBA1 and PBA2. To prevent hybridization of PBA1 and PBA2 in the absence of PDGF-BB, PBA1 is pre-hybridized with B-DNA before starting the assay. Hence, in the absence of the target protein, the addition of *N*-methylmesoporphyrin IX (NMM), a fluorescent dye which is also a strong G4-binder, leads to low fluorescent emission. On the contrary, in the presence of PDGF-BB, the interaction of PBA1/B-DNA and PBA2 with the target results into an increase of the local concentration of the G4-forming sequences, therefore promoting bimolecular G4s formation and B-DNA strands release. Finally, addition of NMM generates an enhanced fluorescent emission, as a consequence of its interaction with the bimolecular G4 (Figure 14a). Though quite elaborate, this assay allows high sensitivity (detection limit of 3.2 nM) and high selectivity, proving to be extremely efficient for PDGF-BB monitoring in diluted human serum samples.

Previously, Zhou et al. [115] have used a different label-free methodology for the detection of PDGF-BB, exploiting the DNA-intercalating cyanine dye 1,1′-(4,4,8,8-tetramethyl-4,8-diazaundecamethylene)-bis-4-[3-methyl-2,3-dihydro(benzo-1,3-thiazole)-2-methylidene] quinolinium tetraiodide, named TOTO. TOTO fluorescence is very low in solution but its fluorescence increases 25 times when bound to the aptamer. Upon addition of PDGF-BB to the aptamer/TOTO solution, a significant and detectable decrease of fluorescence is observed due to the release of the bound TOTO as a consequence of the aptamer conformational change induced by the protein (Figure 14b). The detection limit of PDGF-BB using this method is 100 pM.

A similar approach has been more recently used by Penmatsa et al. [116], who covalently immobilized the PDGF-BB aptamer on a 3D carbon microarray platform and exploited the above described detection method based on the release of bound TOTO from the aptamer. This sensor shows a linear dependence of the fluorescence intensity on the protein concentration in the sub-nanomolar range with a detection limit of 5 pM.

Another label-free system has been designed by Wang et al. [117], based on the enzyme-free hybridization chain reaction (HCR) [164,165,166], employing three DNA probes, denoted as HP (helper DNA probe), H1 (hairpin probe 1) and H2 (hairpin probe 2). HP is constituted by the PDGF-BB aptamer, a sequence complementary to H1 at its 5′-end and an A_10_ linker, while H2 is a sequence complementary to H1 (Figure 14c). Graphene oxide (GO) surface and the fluorescent dye SYBR Green I (SG) are also used in this detection strategy. In the absence of the target protein, HP, H1 and H2 form stable unimolecular hairpin duplex structures and can be free in solution or adsorbed, as well as SG, on the GO surface, thus resulting in a weak fluorescence signal. In the presence of PDGF-BB, HP recognizes the target through its aptameric sequence and changes its conformation, thus exposing the part of its sequence complementary to H1, which can therefore hybridize to HP; in parallel, the denaturation of the H1 hairpin duplex allows the hybridization of H2 with H1. The opening of the H2 hairpin duplex exposes the H2 terminal that can interact with another H1 molecule and so on, thus triggering the enzyme-free HCR. The obtained long chain of target-HP/(H1/H2)_n_ interacts with multiple SG molecules producing a strong fluorescent signal. Moreover, the GO surface is able to adsorb free H1, H2 and SG and not the long chain of HP, thus increasing the signal/noise ratio (Figure 14c) [167]. The detection limit for this assay is 1.25 pM, allowing the highest sensitivity for PDGF-BB detection compared to the other reported methods and good specificity even in complex matrices.

A simpler probe exploiting a fluorescently-labelled aptamer for the PDGF detection has been designed by Fang et al. [118]. Indeed, they functionalized the PDGF-BB aptamer with the couple 6-amino fluorescein/dabcyl at its 5′- and 3′-ends, respectively as fluorophore/quencher pair (Figure 14d). In solution, in the absence of PDGF-BB, the fluorophore and the quencher are far apart and a fluorescence signal is observed, while in the presence of the target the two termini are in close proximity and fluorescence quenching is obtained, thus allowing to detect PDGF-BB at 110 pM, even in serum and in cell culture media.

Another method for the detection of PDGF-BB exploiting fluorescently-labelled aptamers has been proposed by Liang et al. [119]. Their strategy is based on monitoring the fluorescence recovery of the dye-labelled aptamer induced by the target protein after quenching by a GO surface. The π-π stacking interactions of the fluorescein labelled-aptamer with GO leads to fluorescence quenching by FRET from dye to GO. Then, when this system is incubated with PDGF-BB, the aptamer-protein complex formation moves away fluorescein from GO, leading to fluorescence recovery (Figure 14e). The detection limit for this aptasensor is estimated to be 167 pM. The method, also effective in real samples analysis, is highly selective for PDGF-BB protein.

A different sensing strategy using labelled aptamers has been developed by Yang et al. who engineered a light-switching excimer aptamer probe [120]. They conjugated the PDGF-BB aptameric sequence with one pyrene residue at each end. In the absence of the target protein, the aptamer has a characteristic open conformation in which the two ends are far apart; in the presence of PDGF-BB, the interaction aptamer-protein causes a conformational change in the aptamer structure, bringing the pyrene moieties close to each other and allowing them to form an excimer (Figure 14f). This event switches the fluorescence emission of the pyrene tags from 400 nm to 485 nm. Furthermore, the longer fluorescence lifetime of pyrene excimer than all the other species present in real samples allows discriminating excimer signal from background interference by time-resolved fluorescence experiments, achieving a detection limit in the picomolar range in few seconds even in cell media. 

The real-time monitoring of the binding between a labelled aptamer and the target protein can be also performed by measuring the fluorescence anisotropy change [168], as realized for example by Fang et al. [121]. A fluorescein-labelled PDGF-BB aptamer has been used as a probe for an easy monitoring of the anisotropy increase produced upon binding to the target protein (Figure 14g). The significant increase in anisotropy is due to the large difference in molecular size between the free fluorescent aptamer and its PDGF-bound complex. This assay has a detection limit of 220 pM.

More recently, Zhang et al. [162] have modified a truncated form of the PDGF-BB aptamer inserting at its 5′-end a fluorescent tetramethylrhodamine. Upon protein binding, the fluorescence anisotropy of the selected aptamer significantly decreases, allowing the detection of PDGF-BB even in the picomolar range [122]. This behaviour, completely opposite to the fluorescence anisotropy increase, typically observed after aptamer-protein interaction, is attributed to the aptamer conformational change that reduces the interactions between the fluorophore and the adjacent nucleobases (Figure 14h).

Labelled PDGF aptamers have been recently used by Zhao et al. also as cell-surface sensors for real-time probing of signalling molecules in the cellular environment [123]. Indeed, they covalently attached the aptamer labelled with two dyes (FAM/dabcyl or Cy3/Cy5) to the membrane of mesenchymal stem cells (MSCs) [169,170]. To allow the binding to the cell surface, the original PDGF-BB aptamer sequence has been extended by a short oligonucleotide complementary to a single strand conjugated with biotin. MSCs have been modified by reacting the cell surface amines with sulfonated biotinyl-*N*-hydroxysuccinimide (NHS-biotin) [171,172]. Finally, the interaction of the biotin-conjugated PDGF extended-aptamer with streptavidin enabled the binding of the sensor to the cell surface. When bound to PDGF, the aptamer changes conformation and the dyes come close to each other, producing, in the quench sensor, a FAM fluorescence quenching by dabcyl, or, in the FRET sensor, a donor dye (Cy3) fluorescence decrease and an acceptor dye (Cy5) fluorescence increase. These sensors recognize PDGF added in cell culture medium, as well as PDGF secreted by adjacent cells, with a detection limit in the picomolar range.

### 2.3. Angiogenin

Angiogenin is a 14-kDa single chain protein, homolog of bovine pancreatic ribonuclease A [173], able to stimulate cell proliferation, migration, adhesion, invasion and playing key roles in tumorigenesis and angiogenesis [174,175,176]. Angiogenin levels are significantly higher in the serum of cancer patients than in healthy humans [175,177,178]. Moreover, the angiogenin levels are reduced in cancer patients undergoing successful treatments, while are increased after tumour recurrence [179,180]. Hence, the evaluation of angiogenin concentration in tumour tissues and serum can be clinically useful for early diagnosis, as well as for monitoring tumour growth and progression.

The angiogenin aptamer has been identified by Nobile et al. in 1998 [181]. From a 72-mer oligonucleotide library of ca. 10^14^ molecules, 19 were selected as the best angiogenin ligands. Among them, one was found to be the most potent inhibitor of angiogenin ribonucleolytic activity. This oligonucleotide was then shortened to a 45-mer, named AL6-B, selecting, after division of secondary structure of the longer oligonucleotide in two lobes, the one with the highest inhibitory effects on angiogenin ribonucleolytic activity. AL6-B is able to inhibit angiogenic and cell proliferative activity of angiogenin without interfering with its nuclear translocation in human endothelial cells.

In 2007/2008, Li et al. have described three different fluorescent probes for the detection of angiogenin in serum samples of healthy or cancer patients [125,126,127]. In the first designed probe [125], they have exploited the fluorescence anisotropy positive change [168] from the free labelled aptamer, in this case AL6-B conjugated with fluorescein at its 5′-end, to the aptamer-protein complex, to monitor the binding event as well as to measure the angiogenin concentration (a similar approach is depicted in Figure 14g). The assay is highly selective with a detection limit of 1 nM and has been also applied in serum samples of lung cancer patients. 

The same group has then proposed a dual-labelled probe based on FRET for rapid angiogenin detection and quantification [126]. Indeed, they have conjugated AL6-B aptamer with the couple 6-carboxyfluorescein/6-carboxytetramethylrhodamine, respectively at its 5′- and 3′-end, as fluorophore donor and acceptor pair (a similar approach is described in Figure 14d). In the absence of the target protein, the aptamer is in equilibrium between a stem-loop secondary structure and a non-structured form [181]. In the presence of angiogenin, only the structured form, in which the donor and the acceptor are in close proximity, is present and enhanced FRET is observed. Due to the high specificity of the aptamer-protein interaction, the system is highly selective toward angiogenin target. This method has a detection limit of 200 pM, is very quick and can be applied in real samples, e.g., healthy and lung cancer patients’ serum samples.

Li et al. have also described a probe for real-time imaging of angiogenin in living cells by using confocal laser scanning microscopy [127]. To this purpose Cy5 has been conjugated to the 5′-end of AL6-B, then tested in cells expressing angiogenin receptors on their membrane (HUVE cells and MCF-7 breast cancer cells) and in control cells not expressing these receptors (COS-7 cells). When aptamer-angiogenin complexes are added to cell cultures, the conjugates selectively bind to HUVE and MCF-7 cells. Confocal microscopy experiments on these systems have also proved that nuclear translocation of angiogenin is very rapid: 2 min are sufficient to enter and 15 min to reach saturation. Furthermore, Li et al. have observed fusion, deformation and migration of HUVE and MCF-7 cultured cells, for the first time directly visualizing angiogenin-induced cell movements upon addition of aptamer-angiogenin complexes.

**Figure 15:**
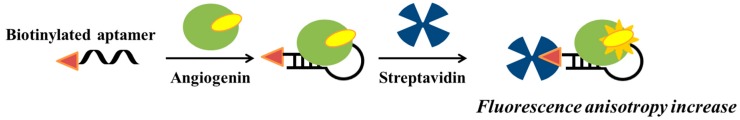
Schematic representation of angiogenin detection strategy based on streptavidin-triggered amplified fluorescence polarization [124].

More recently, Choi et al. have proposed the use of streptavidin-triggered amplified fluorescence polarization (SAFP) for the detection of angiogenin [124]. Angiogenin has been fluorescently-labelled with Alexa Fluor 488, while the angiogenin aptamer has been conjugated with biotin at its 5′-end (Figure 15). In the absence of streptavidin, the interaction between the fluorescently labelled-protein and the biotinylated aptamer leads to a low fluorescent polarization signal, while in the presence of streptavidin the signal is strongly amplified, allowing the detection of angiogenin with a limit of 6.3 nM.

### 2.4. Mucin 1

Mucins are a family of high molecular weight and heavily *O*-glycosylated proteins classified into two subfamilies, cell surface associated and secreted (gel-forming) mucins [182]. Mucin 1 (MUC1) is one of the transmembrane mucins, with a heavily glycosylated extracellular domain that extends up to 200–500 nm from the cell surface, highly expressed in malignant cancers such as breast, ovarian, lung, pancreatic and prostate cancers [183,184,185]. In particular, overexpression and under glycosylation of MUC1, together with relocalization to the entire cell surface, have been reported for almost all epithelial carcinomas, including 90% of breast cancers. Therefore, MUC1 is a marker for poor prognosis in different types of cancers, as well as an ideal target for imaging because of its direct accessibility on membrane cells [186]. 

Several high affinity aptamers have been isolated against MUC1: in particular, most of them have been selected against the exposed peptide backbone, named variable number tandem repeats (VNTR) peptide, containing highly immunogenic epitopes [187], or against the Tn (GalNAc) and T (Galβ1-3GalNAc) antigens *O*-linked to the peptide tandem repeat (for a detailed review, see reference [188]). Many of these aptamers are efficiently and specifically internalized through receptor-mediated endocytosis by epithelial cancer cells and can be used as delivery vehicles to specifically direct pro-drug cargoes, e.g., in photodynamic therapy (PDT) [189], or other cytotoxic agents (doxorubicin, paclitaxel, epirubicin, specific siRNA) [190,191,192] into cells to visualize but, above all, kill them.

To date, among the various aptamers identified against MUC1 [188], only the aptamer selected by Ferreira et al. has been used in fluorescence sensing strategies [187].

In the most recent application of Ferreira’s aptamer, Ma et al. have developed a HCR-based assay for the detection of MUC1 peptide [128]. In the assay, three oligonucleotide sequences are used: the MUC1 aptameric sequence, elongated with a complementary sequence in order to form a stable unimolecular hairpin duplex and two hairpin probes, H1 and H2, labelled with FAM at the 3′- and 5′-end, respectively (a similar approach is described in Figure 14c). Upon interaction with the target protein, the hairpin structure of the aptamer is opened, initiating the chain reaction in which H1 and H2 are involved. Furthermore, the subsequent addition of graphene oxide allows the adsorption of free H1 and H2, thus resulting in background reduction. The detection limit of the method is 3.33 pM and the optimal reaction time for HCR is 1 h. 

Graphene oxide is used also in the method described by He et al. [129]. They exploited GO as a surface to quench the Cy5-conjugated MUC1 aptamer fluorescence. Only in the presence of MUC1, the fluorescence is recovered after detachment from the surface of the labelled aptamer forming a complex with the protein (a similar approach is described in Figure 14e). Exploiting this method, MUC1 can be revealed, even in real serum samples, with a detection limit of 28 nM.

### 2.5. VEGF

Vascular endothelial growth factor (VEGF) plays key roles as regulator of angiogenesis and vascular permeabilization [193]. Among the several isoforms of VEGF, VEGF_165_ is the most abundant and potent pro-angiogenic one, typically overexpressed in several cancer types and therefore identified as an important serum biomarker for cancer [194,195].

**Figure 16:**
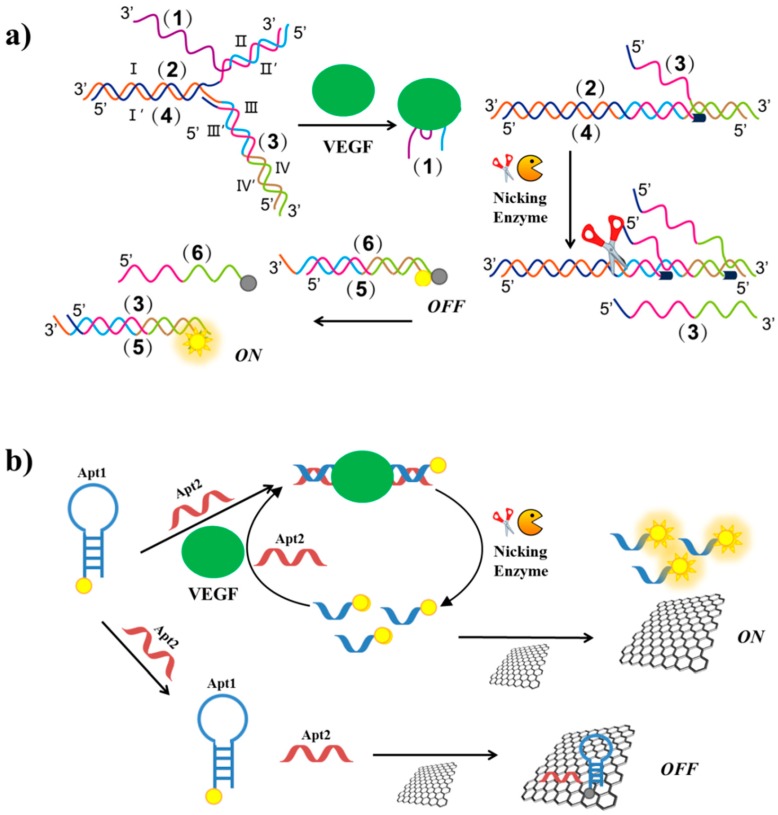
Schematic representation of VEGF detection strategies exploiting nicking endonuclease enzymes, based on DNA assembly structure switching and isothermal amplification after enzyme cleavage (**a**) and VEGF aptamer splitting into two subunits and fluorescence enhancement after target recognition and enzyme cleavage (**b**). Figure 16a is adapted from reference. [132].

In order to target VEGF_165_ in serum samples, Li et al. have developed a strategy based on DNA assembly structure switching and isothermal amplification [132]. Their method exploited six different DNA sequences: the VEGF aptamer (1), (for a recent review, see reference [94]) a primer (2), a protector (3), a template (4), a fluorescent labelled- (5) and a quencher labelled-strand (6). In the absence of VEGF, (1), (2), (3) and (4) strands hybridize forming a complex assembly, which prevents strand displacement and extension reaction (Figure 16a). In the presence of the target, the aptamer sequence is released from the assembly, the II’ domain of (2) is exposed and can interact with III’ domain of (4), displacing the III domain of (3). The formation of the (2)/(4) complex triggers the polymerization reaction mediated by polymerase and deoxynucleotide triphosphates. Upon polymerization, a nicking endonuclease cleaves the duplex (2)/(4) at its specific recognition site, triggering the next displacement cycle, nicking and polymerization reactions. Finally, DNA (3), generated at each cycle, displaces dabcyl labelled-(6) strand from the duplex (5)/(6). After removal of (5) from (6), the FAM dye fluorescence is recovered and a fluorescent signal can be detected (Figure 16a). The detection limit of this method is estimated to be 3.5 pg/mL.

Another method exploiting nicking endonuclease has been developed by Li et al. [133]. The VEGF aptamer is split into two subunits: Apt1 and Apt2 (Figure 16b). Apt1 is a FAM-labelled hairpin duplex containing the cleavage site for the nicking enzyme, while Apt2 is a single strand complementary to Apt1. In the absence of the target, both the oligonucleotides are adsorbed on a GO surface and the dye fluorescence is quenched. In the presence of VEGF, Apt2 and the protein interact with Apt1, thus obtaining the cleavage site which is recognized by the nicking enzyme cleaving Apt1 in two single strands. The short labelled-strand derived from Apt1 weakly interacts with the GO surface and thus a strong fluorescent signal can be detected (Figure 16b). Furthermore, after the endonuclease cleavage, each target can act through many cycles, thus leading to fluorescence amplification. The detection limit is 1 pM and the method has been applied to real biological samples with satisfactory results.

Mita et al. have designed a bound/free separation system for VEGF quantification with a detection limit of 25 nM [134]. In this assay, the fluorescein-labelled aptamer is first incubated with VEGF and then with peptide nucleic acid (PNA)-functionalized beads. In the absence of the target, the aptamer is captured by its complementary PNA on the beads, leading to a fluorescence intensity decrease; in the presence of VEGF, the aptamer bound to the target is not able to hybridize with PNA sequences, thus resulting in a fluorescence intensity increase of the supernatant.

Wang et al. have exploited fluorescence polarization for the detection of VEGF by using a FAM-labelled VEGF aptamer [135]. In the absence of the target, the aptamer is present as a flexible single strand showing low fluorescence anisotropy value, while upon VEGF recognition the aptamer folds into a G4 structure (a similar approach is described in Figure 14g). The interaction G4-VEGF leads to a significant increase in fluorescence anisotropy, thus allowing the VEGF quantification with a detection limit of 320 pM.

The same group has also proposed an alternative method for VEGF detection, based on graphene oxide and FRET [136]. They exploited GO as a surface to quench the labelled-VEGF aptamer fluorescence (a similar approach is described in Figure 14e). Only in the presence of the target protein, the quenched fluorescence is restored as a consequence of the detachment from the surface of the fluorescent aptamer, forming a complex with the protein. This method has a detection limit of 250 pM and proved to be highly specific towards VEGF.

### 2.6. Elastase

Neutrophils are the first line of defence of the innate immune system against infectious agents or non-self-substances, also involved in acute and chronic inflammation [196]. The human neutrophil elastase (HNE), a serine proteinase, is the main enzyme released from neutrophil granulocytes [197]. HNE is also involved in cancer progression, enhancing tumour proliferation and metastasis [198]. In addition, HNE levels increase during tumorigenesis [199] and therefore HNE can be considered a good cancer biomarker.

In 1997 Charlton et al. identified an aptamer against HNE secreted from activated neutrophils by selection from a randomized DNA-valine phosphonate library [200]. The same research group used this aptamer, after suitable fluorescence labelling, for in vitro and in vivo diagnostic experiments [137]. In vitro studies proved that the fluorescence intensity of the aptamer well correlated with the level of elastase activity; furthermore, in vivo experiments proved the aptamer efficacy in visualizing inflammation in rats due to its good target-to-background ratio and fast clearance.

**Figure 17:**
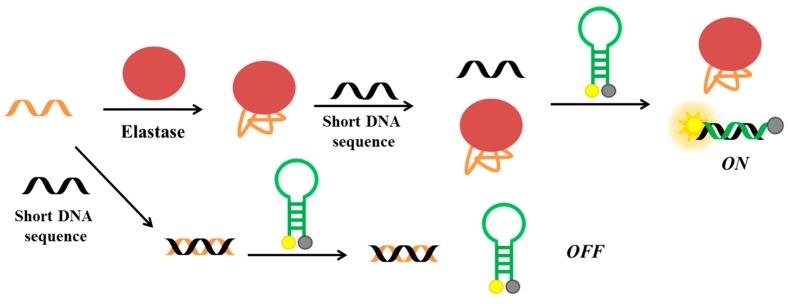
Schematic representation of elastase detection strategy based on competitive-binding involving the elastase aptamer, a molecular beacon and a short DNA sequence [138].

More recently, He et al. have developed a fluorescent aptasensor based on competitive-binding for the detection of HNE [138]. In this assay, three oligonucleotides are used: the HNE aptamer, a molecular beacon conjugated with fluorescein and dabcyl at its 5′- and 3′-end, respectively, and a short DNA sequence, complementary to part of the aptamer sequence and to the loop of molecular beacon (Figure 17). The aptamer is first incubated with the short DNA and then with the molecular beacon. Therefore, in the absence of the target, the molecular beacon is not able to interact with the short DNA, since the latter is already bound to the aptameric sequence. In the presence of HNE, the aptamer binds the protein and not the short DNA, which can therefore hybridize the molecular beacon, thus producing a strong fluorescent signal as a result of the increased distance between the fluorescent and the quencher. The detection limit for this method is estimated to be 47 pM.

### 2.7. PTK7

Protein tyrosine kinase 7 (PTK7) is a member of receptor protein tyrosine kinase-like molecules, which is involved in tumorigenesis, cell motility, invasion and metastasis [201,202,203,204]. 

Recent studies have shown that overexpression of PTK7 is associated with cancer risk and that PTK7 can be used as a biomarker for predicting poor prognosis [205]. 

Wang et al. have developed a theranostic approach based on a structure-switching aptamer able to trigger HCR on the cell surface [139]. In this strategy, an extended sequence of the PTK7 aptamer (Sgc8) [206,207] able to form a unimolecular hairpin duplex and two hairpin probes (H1 and H2) are used (a similar HCR approach is described in Figure 14c). H1 is labelled with Cy5 fluorophore and BHQ3 quencher, while H2 is conjugated with a cisplatin prodrug [208]. Only in the presence of cells exposing PTK7, as a consequence of the interaction with the target protein, the aptamer switches its conformation and HCR, involving also H1 and H2, can initiate. Thus, a fluorescent signal is generated since the fluorophore and the quencher of H1 cannot interact. Furthermore, the HCR product, delivering the antitumoral prodrug, is accumulated and internalized only into cells expressing PTK7. Therefore, this method can be applied for selective tumour therapy and diagnosis allowing a detection limit of 1 pM.

More recently, Calzada et al. have developed a different fluorescent aptamer for PTK7 detection [140]. In particular, they used a truncated sequence of the original aptamer Sgc8 (Sgc8c) [206], modified at its 5′-end with an amino-derivatized 6-carbon spacer conjugated to a NIR fluorophore (Alexa Fluor 647). This aptamer binds melanoma and lymphoma cell lines in vitro, while in vivo studies in tumour-bearing mice have showed a good uptake and rapid clearance.

### 2.8. Lysozyme

Lysozyme is a small enzyme playing crucial roles in the innate immune system [209,210]. Moreover, several studies have proved that lysozyme levels in saliva and serum can be used as prognostic factors in patients with breast cancer or monocytic and monomyelocytic leukaemia [211,212,213]. Taking into account the relevance of lysozyme as a cancer biomarker, Zou et al. have developed a simple and sensitive method based on fluorescence anisotropy [144]. A specific FAM-labelled lysozyme aptamer produces fluorescence anisotropy increase upon protein binding [214], thus allowing the detection in real-time of the binding events (a similar approach is described in Figure 14g). The limit of detection proved to be 4.9 nM, with satisfactory results obtained with real salivary samples.

**Figure 18:**
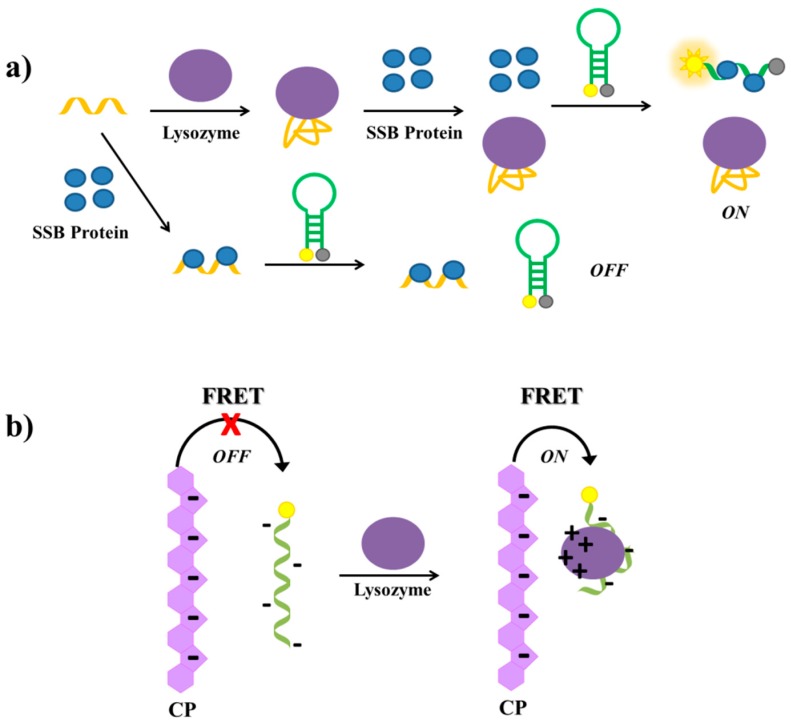
Schematic representation of lysozyme detection strategies based on: (**a**) competitive binding involving the lysozyme aptamer, a molecular beacon and single-stranded binding (SSB) protein [141]; (**b**) FRET between an anionic conjugated polymer (CP) and the labelled lysozyme aptamer [145].

Tang et al. have described a label-free aptamer-based strategy for the selective detection of proteins and proved its feasibility on lysozyme [141]. The lysozyme aptamer [215] is first incubated with single-stranded binding (SSB) protein and then with a molecular beacon equipped with a fluorophore and a quencher at its termini (Figure 18a). In the absence of lysozyme, the aptamer binds the SSB protein. Therefore, the subsequently added molecular beacon is not able to interact with the SSB protein and maintains its hairpin structure, not producing a significant fluorescence signal. In the presence of lysozyme, the aptamer binds its target protein; therefore, the free SSB protein can bind the molecular beacon, disrupting its secondary structure and generating a strong fluorescence signal due to the spatial separation of the fluorescent and the quencher (Figure 18a). The detection limit of this assay, used also to detect lysozyme in human saliva samples with good results, proved to be 200 pM.

More recently, Chen et al. have proposed an exonuclease III-based amplification assay to detect lysozyme [142]. In this method, exonuclease III, a graphene oxide surface, a hairpin probe (HP) and a signal probe (SP) are used (a similar approach is described in Figure 16b). HP contains the lysozyme aptameric sequence [216] and a region complementary to a part of the aptameric sequence as well as to a part of SP. Therefore, HP is able to form a unimolecular hairpin duplex secondary structure. The signal probe is a FAM-labelled oligonucleotide. In the absence of lysozyme, SP is not able to hybridize its complementary strand on HP since the latter is involved in the formation of the hairpin structure. As a consequence, exonuclease is not able to digest SP which is adsorbed on the GO surface and FAM fluorescence is quenched. In the presence of lysozyme, the interaction between the protein and the aptamer results in the exposition of the HP region complementary to SP, thus a duplex structure is formed and the exonuclease can digest SP. Upon addition of GO, the digested short DNA fragments and the liberated FAM dyes are not adsorbed on GO and a fluorescent signal is observed, with a detection limit of 0.125 µg/mL.

Another approach to detect lysozyme in human serum has been proposed by Huang et al. [143]. This method, based on a competition-mediated pyrene-switching aptasensor, exploits two DNA sequences: the lysozyme aptamer [215] and a dual-pyrene labelled hairpin, complementary to a part of the aptamer. In the absence of lysozyme, the two DNA sequences are hybridized and the pyrene moieties are not in close proximity; in the presence of lysozyme, the aptamer is captured by the protein and the dual-pyrene labelled sequence can fold into a hairpin structure. In this way, the pyrene tags come into close proximity to form an excimer, resulting in a detectable fluorescence emission shift (a similar approach is described in Figure 14f). The detection limit of this method is 200 pM.

Another aptamer-based assay for the detection of lysozyme has been reported by Wang et al. [145], who have developed a strategy based on FRET between an anionic conjugated polymer (CP) and the FAM-labelled lysozyme aptamer [215]. The polymer and the aptamer are both negatively charged, thus they repel each other in solution and no FRET is observed. In the presence of lysozyme, a complex between the aptamer and the protein is formed. At physiological pH, this complex, differently from the free aptamer, has positive surface charges able to interact with the anionic conjugated polymer, thus bringing the CP and the complex in close proximity, which generates FAM energy transfer and light up (Figure 18b). The detection limit of this method is estimated to be 0.80 µg/mL.

A brief summary of the main features of the here discussed aptamer-based strategies is reported in Table 1.

### 2.9. Cancer Cells

Visualization of cancer cells requires discriminating malignant from healthy cells by objective criteria with high specificity. The possibility to identify tumour markers expressed on the surface of cancer cells with suitable imaging probes has revolutionized cancer diagnosis, also offering an exceptional tool in surgery for tumour ablation [217,218]. In this context, fluorescent aptamers can be used as effective tools to detect whole cancer cells by exploiting the highly specific binding to the extracellular domains of their targets exposed on the cell surface. In this case, the target acts as a cellular biomarker for highly sensitive cancer cells imaging/detection.

Therefore, different types of fluorescent aptamers have been developed for the in vitro and in vivo imaging of tumour cells and tissues, allowing a significant improvement in prognosis and even in the early treatment of cancer [219,220]. The efficiency of the aptamer-based spectrofluorometric assays in the sensitive detection of cancer cells can be further improved by association with biocompatible nanostructures, as evidenced by the successful application of aptamer-conjugated fluorescence silica nanoparticles [221], carbon nanodots [222,223] or metal nanoparticles [224] for cancer cells detection.

The first application of fluorescent aptamers generated from cell-SELEX for in vivo fluorescence imaging has been reported by Shi et al. to identify tumour cells in mice. A Cy5-labeled aptamer (Cy5-TD05, specific for Ramos, a B-cell lymphoma) has been used for fluorescence imaging in Ramos tumour-bearing xenograft nude mice allowing the direct monitoring of the spatial and temporal distribution of the aptamer [225]. After intravenous injection, whole-body fluorescence imaging demonstrated that Cy5-TD05 effectively recognized Ramos tumours with high sensitivity and selectivity.

The fluorophore Cy5 has been successfully exploited also for the derivatization of the aptamer, named S6, identified via cell-SELEX against A549 lung carcinoma cells. The fluorescently-labelled aptamer Cy5-S6 specifically recognized A549 carcinoma cells in both buffer and serum settings as determined by flow cytometry assays [226]. Furthermore, Cy5-S6 proved to be effective also for in vivo fluorescence imaging, selectively identifying A549 tumours when intravenously injected into nude mice.

Among the aptamers developed against specific proteins validated as cancer targets, those against nucleolin, mucin and protein tyrosine kinase 7 are the most widely used for in vitro and in vivo experiments.

The G-quadruplex-forming AS1411 is a nucleolin-targeting aptamer, which has entered phase I/II clinical trials for the potential treatment of different types of cancer [227]. In a recent study, a cellular confocal microscope assay proved that AS1411 conjugated with the fluorescent ligand protoporphyrin IX (PPIX) allowed in situ labelling and imaging of nucleolin-overexpressing HeLa cancer cells, discriminating them from normal cells in human serum [228].

**Figure 19:**
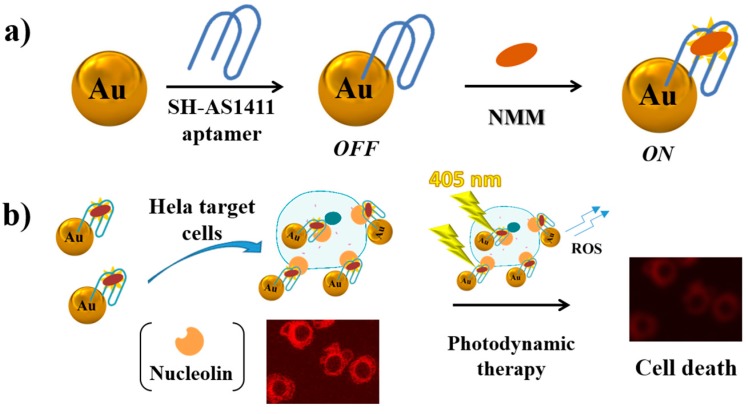
Schematic representation of (**a**) preparation of AS1411-functionalized gold nanoparticles bound with fluorescent *N*-methylmesoporphyrin IX (NMM) and (**b**) possible use of this system for both cancer cell imaging and photodynamic therapy, as described in reference [229].

In a multivalent approach, AS1411-functionalized gold nanoparticles bound with fluorescent *N*-methylmesoporphyrin IX (NMM, Figure 19a) have been successfully applied for both cancer cell imaging and photodynamic therapy [229]. Generally, the porphyrin derivatives are weakly fluorescent as such in aqueous solution, while exhibit a dramatic fluorescence enhancement upon binding to a G-quadruplex DNA. Porphyrin derivatives can be also used as photosensitizers in photodynamic therapy (PDT) of cancer, producing upon irradiation highly cytotoxic reactive oxygen species (ROS) which kill tumour cells. This strategy has been successfully combined with AS1411-functionalized gold nanoparticles and applied to detect HeLa cancer cells, destroying them after white light irradiation of the nanoparticles (Figure 19b) [229].

Three types of MUC1 aptamer-conjugated Rubpy-doped silica nanoparticles (SiNPs) have been prepared by Cai and co-workers for MCF-7 cells (MUC1 positive) recognition and labelling, detected by fluorescence microscopy [230]. Among these probes, the highest binding efficiency for MUC1 has been observed for aptamer-SiNPs containing a flexible polyethylene glycol (PEG) linker (8–12 units) bridging the aptamer and the NPs. The number of aptamers per nanoparticle has been increased employing biotinylated oligonucleotides and PEG-avidin nanoparticles and exploiting the high efficiency in biotin-avidin interactions. 

In vivo tumour targeting monitored by fluorescence imaging has been also achieved using a NIR fluorescent dye-labelled MUC1 aptamer [131], in which one end of a PEG spacer has been linked to the NIR fluorescent hydrophilic cyanine dye and the other coupled to the aptamer. The PEG-modified probe preferentially accumulated in MUC1 positive cells (e.g., MCF-7) showing improved contrast for MUC1 positive tumours in vivo. 

Another MUC1 aptamer, labelled with Cy3 dye, has been investigated in 3D cell aggregates (multicellular spheroids of MCF-7 cells) and compared to an anti-MUC1 antibody for its capacity to visualize cancer cells [130]. Confocal microscopy experiments proved that, while anti-MUC1 antibody interacted only with the protein target at the surface of the spheroid, Cy3-MUC1-aptamer was able to penetrate inside these 3D tumour models and internalize into the cancer cells.

Despite several “always-on” fluorescent aptamer probes have been successfully produced as imaging agents for cancer cells, they may suffer from undesired background and limited contrast. To address this problem, thanks to the possibility to chemically modify aptamers, different activatable aptamer-based probes (AAPs) have been produced for targeting membrane proteins of cancer cells, obtaining a more effective contrast-enhanced cancer visualization in animal models based on FRET. These AAPs typically display a quenched fluorescence in their free form but upon binding to the target undergo a conformational change with fluorescence activation.

**Figure 20:**
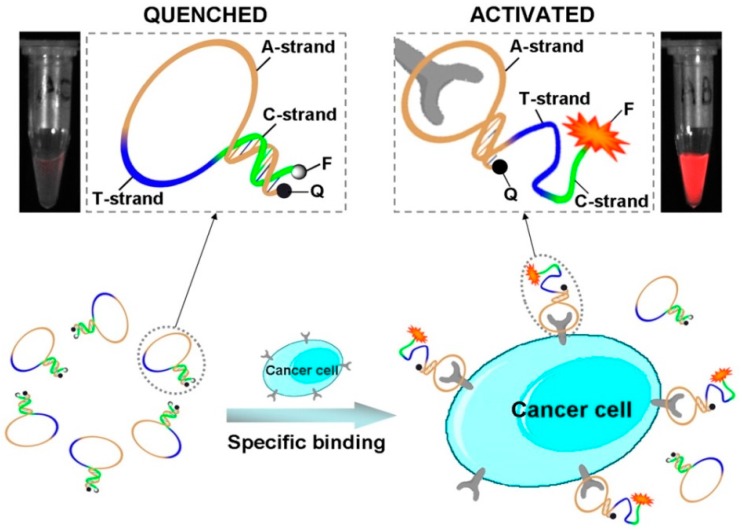
Schematic representation of activatable aptamer probe (AAP)-based strategy for in vitro and in vivo cancer imaging exploiting a cell membrane protein-triggered conformation change. Figure reproduced with permission from reference [231]. Copyright 2011 PubMed Central (PMC).

A proof-of-concept study has been carried out on CCRF-CEM cells (human acute lymphoblastic leukaemia T cells which overexpress protein tyrosine kinase 7 (PTK7)) using AAPs based on a conformation-switchable sgc8c aptamer, targeting PTK7 [207], labelled with a fluorophore/quencher pair [231]. This AAP is a single-stranded oligonucleotide consisting of three fragments: a cancer-targeted aptamer sequence (A-strand), a poly-T linker (T-strand) and a short DNA sequence (C-strand) complementary to a part of the A-strand, with a FAM fluorophore and a BQH1 quencher attached at the ends. In the absence of the target, the AAP is structured in a hairpin form, resulting in a quenched fluorescence. When interacting with the protein receptor on the surface of target cancer cells, the aptamer conformation is altered, driving the dye far from its quencher, which results in fluorescence emission (Figure 20). This sgc8-based AAP is specifically activated by CCRF-CEM cells, showing fluorescence enhancement and good sensitivity (improved signal/background ratio) also in the detection of CCRF-CEM tumour sites in mice.

Compared to always-on aptamer probes, the AAPs considerably minimized the background signal from non-target tissues, resulting in significantly enhanced image contrast and shortened diagnosis time.

**Figure 21:**
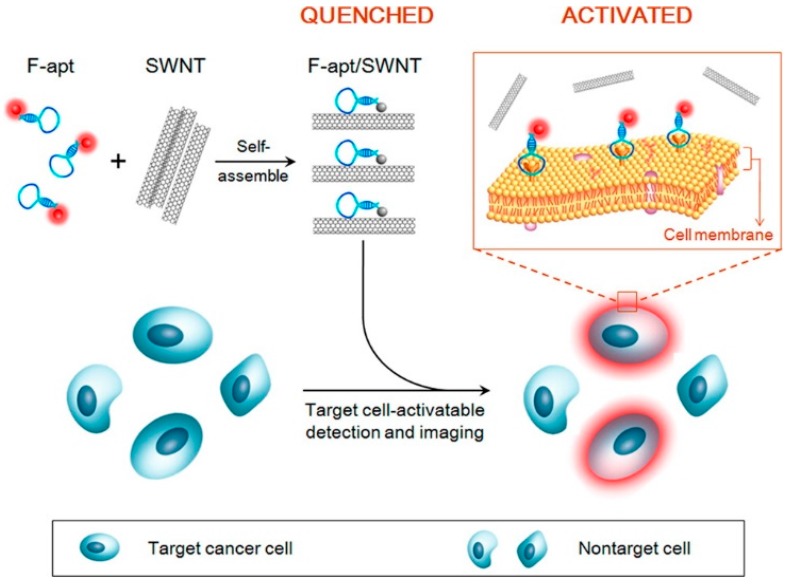
Schematic representation of the activatable fluorescence probing strategy for target CCRF-CEM cancer cells based on the self-assembly between a fluorophore-labelled aptamer (Cy5-labeled sgc8c) and single-walled carbon nanotubes (SWNT). Figure reproduced with permission from reference. [232]. Copyright © 2014 American Chemical Society.

The production of AAPs usually involves a complex design and synthesis and their stability in biological systems is not always satisfactory. In an effort to overcome these limitations, a simpler strategy has been proposed by Yan and co-workers for cancer cell probing based on fluorophore-labelled aptamer/single-walled carbon nanotube (SWNT) ensembles [232]. In this system, single-walled carbon nanotubes (SWNT) have been used as aptamer carriers and fluorescence quenchers, whereas Cy5-labeled sgc8c activatable aptamers acted as recognition probes and fluorescent reporters for in vitro CCRF-CEM cancer cell detection and in vivo cancer imaging. Cy5-labeled sgc8c aptamers conjugated through π-π stacking interactions with SWNT showed a quenched fluorescence signal, so, when injected into tumour-bearing mice, they remained optically silent until reaching the tumour. In the presence of tumour sites, the Cy5-labeled sgc8c aptamers detached from SWNT and bound to specific biomarker of CCRF-CEM cancer cells, thus resulting in dramatically enhanced fluorescence (Figure 21). This versatile activatable aptamer-fluorescence probing platform based on aptamer/carbon nanotube ensembles had significantly improved fluorescence signal-to-noise ratio in tumour-bearing mice.

In a similar example, AS1411, the nucleolin aptamer, has been wrapped around water-soluble fluorescent carbon dots and used as a probe for the detection of several types of cancer cells. Mouse breast 4T1, human breast MCF7 and human cervical HeLa cancer cells have been selected as target cells, while human foreskin fibroblast cells HFFF-PI6 served as control cells. The specific targeting of cancer cells by AS1411 aptamers caused their release from carbon dots and enhanced the fluorescence intensity (signal-on method) [233].

**Figure 22:**
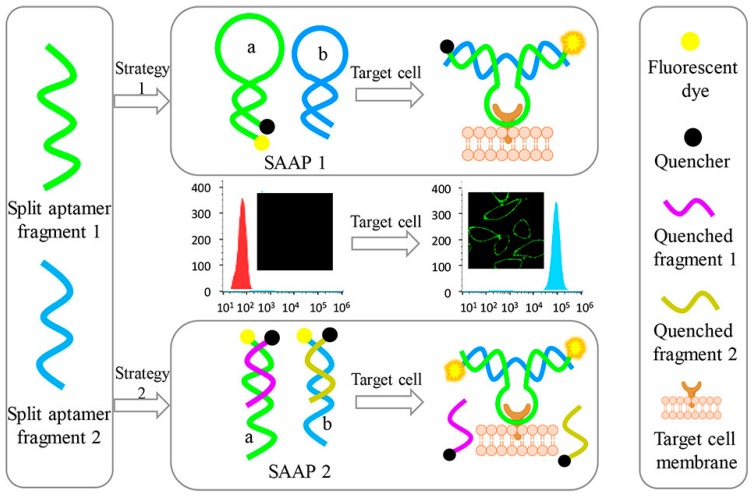
Schematic representation of two different self-assembly and activation strategies for cancer cell detection. Figure reproduced with permission from reference [234]. Copyright © 2017 American Chemical Society.

Another interesting example of activatable aptamers for sensitive detection of cancer cells with high signal-to-background ratio is based on a self-assembling activatable probe (SAAP) with split aptamers [234]. The SAAP probe is designed to give initial quenched fluorescence for the close proximity of the FAM fluorophore and the BQH1 quencher; only in the presence of target cells the split aptamers self-assemble and thus activate fluorescence by intramolecular and intermolecular fluorescence quenching strategies. For the proof of concept, a DNA aptamer ZY11 developed through cell-SELEX against target hepatocellular carcinoma SMMC-7721 (7721) cells has been selected. This aptamer, with two stems and one recognition loop, has been carefully split into two fragments and employed in two different strategies (Figure 22). With these approaches the signal-to-background ratio raised to ∼40 achieving a very low detection limit (7 target 7721 cells in 100 μL of binding buffer) [234]. Remarkably, this method performs well for detecting target cells in 50% human serum and cell samples, demonstrating high potential in cancer therapy and diagnosis.

## 3. Conclusions

Notwithstanding the continuous efforts and increased enthusiasm of the scientific community for aptamers, the gap between potential of DNA/RNA aptamers and their practical applications into clinics is still far from being bridged. The reasons why aptamers have not reached the expected success in both therapy and diagnostics are manifold. Among others, exclusive intellectual property protection for SELEX technology and insufficient development pathway, investment as well as related knowledge still limit the expansion of aptamers, thus favouring antibodies, for which a well-developed commercial infrastructure has been established. 

In this context, the possibility to exploit aptamers in connection with suitable fluorescent probes responsive to the binding events, thus obtaining unique information about their effective targeting, is an exceptional, major advantage over antibodies, which can give more impetus to the development of this promising class of therapeutic and diagnostic tools.

Remarkably, aptamers can be combined not only with fluorescent systems but also with a variety of nanocarriers, in which one or more drugs can be also embedded, being internalized in a cell-specific manner upon binding to cellular receptors. This strategy could provide the basis for synergistic therapeutic strategies in which two or more drugs are simultaneously administered in a combinatorial therapy format, of particular interest, e.g., in bacterial and viral treatment, cancer immunotherapy, targeted drug delivery as well as for tumour precise localization and ablation.

Aptamer-based therapeutics, equipped with extremely sensitive and environment-responsive fluorescent probes, can thus be a good option to obtain superior biological functions and pharmacokinetic profiles in theranostic applications.

## Figures and Tables

**Table 1:** Summary of the detection strategies for the here reported cancer-related protein targets.

Target	Labelling Method	Fluorophore ^a^/Quencher ^b^	Fluorescent Dye ^c^	Mode	Sample	Limit of Detection	Reference
Thrombin	Label-free	--	Ir(III) complex	Signal-ON	serum, urine, saliva	0.05 pM	[97]
	Label-free	--	hemin	Signal-ON	buffer solution	1 pM	[109]
	Label-free	--	SYBR Gold	Signal-ON	serum	680 nM	[110]
	Covalent	6-carboxyfluorescein/dabcyl	--	Signal-OFF	buffer solution	112 pM	[98]
	Covalent	dansyl/β-cyclodextrin	--	Signal-ON	buffer solution	n.d.	[99]
	Covalent	fluorescein/dabcyl	--	Signal-ON	buffer solution	0.2 μM	[102]
	Covalent	fluorescein/dabcyl	--	Signal-ON	buffer solution	3 μM	[103]
	Covalent	pyrrolo dC	---	Signal-ON	buffer solution	10 μM	[70]
	Covalent	6-carboxyfluorescein/Iowa Black FQ	--	Signal-OFF	buffer solution	10 μM	[70]
	Covalent	6-carboxytetramethylrhodamine/AuNP	--	Signal-ON	buffer solution	0.14 nM	[104]
	Covalent	Cy3/AuNP		Signal-ON	buffer solution	10 pM	[105]
	Covalent	6-carboxyfluorescein/SWCT	--	Signal-ON	buffer solution	1.8 nM	[106]
	Covalent	6-carboxyfluorescein/nano-C_60_	--	Signal-ON	serum	1 nM	[107]
	Covalent	6-carboxyfluorescein/graphene	--	Signal-ON	serum	31.3 pM	[68]
	Covalent	Cy3/MNP	--	Signal-ON	serum	0.5 nM	[108]
	Covalent	Quantum dots/dabcyl	--	Signal-ON	buffer solution	1 μM	[111]
	Covalent	fluorescein/dabcyl	--	Signal-OFF	cell extracts, plasma	1 nM	[112]
	Covalent	6-carboxyfluorescein/ Black Hole Quencher 1	--	Signal-ON	serum	100 pM	[113]
PDGF	Label-free	--	NMM	Signal-ON	serum	3.2 nM	[114]
	Label-free	--	TOTO	Signal-OFF	buffer solution	100 pM	[115]
	Label-free	--	TOTO	Signal-OFF	buffer solution	5 pM	[116]
	Label-free	--	SYBR Green I	Signal-ON	serum	1.25 pM	[117]
	Covalent	6-amino fluorescein/dabcyl	--	Signal-OFF	serum, cell culture media	110 pM	[118]
	Covalent	fluorescein/graphene oxide	--	Signal-ON	serum	167 pM	[119]
	Covalent	pyrene	--	Fluorescence emission shift	cell culture media	pM range	[120]
	Covalent	fluorescein	--	Fluorescence anisotropy increase	buffer solution	220 pM	[121]
	Covalent	tetramethylrhodamine	--	Fluorescence anisotropy decrease	buffer solution	pM range	[122]
	Covalent	fluorescein/dabcyl	--	Signal-OFF	cell culture media	pM range	[123]
Angiogenin	Label free	--	Alexa Fluor 488	Fluorescence anisotropy increase	buffer solution	6.3 nM	[124]
	Covalent	fluorescein	--	Fluorescence anisotropy increase	serum	1 nM	[125]
	Covalent	6-carboxyfluorescein/6-carboxytetramethyl-rhodamine	--	Signal-ON	serum	200 pM	[126]
	Covalent	Cy5	--	Signal-ON	cell culture media	n.d.	[127]
Mucin	Label-free	--	fluorescein	Signal-ON	serum	3.33 pM	[128]
	Covalent	Cy5/graphene oxide	--	Signal-ON	serum	28 nM	[129]
	Covalent	Cy3	--	Signal-ON	cell culture media	n.d.	[130]
	Covalent	MPA	--	Signal-ON	cell culture media, nude mice	n.d.	[131]
VEGF	Label-free	--	6-carboxyfluorescein	Signal-ON	serum	3.5 pg/mL	[132]
	Covalent	fluorescein	--	Signal-ON	buffer solution	1 pM	[133]
	Covalent	fluorescein	--	Signal-ON	buffer solution	25 nM	[134]
	Covalent	6-carboxyfluorescein	--	Fluorescence anisotropy increase	buffer solution	320 pM	[135]
	Covalent	6-carboxyfluorescein	--	Signal-ON	serum	250 pM	[136]
Elastase	Covalent	fluorescein	--	Signal-ON	cell culture media, rats	n.d.	[137]
	Covalent	--	fluorescein	Signal-ON	buffer solution	47 pM	[138]
PTK7	Label-free	--	Cy5	Signal-ON	cell culture media	1 pM	[139]
	Covalent	Alexa Fluor 647	--	Signal-ON	cell culture media, nude mice	n.d.	[140]
Lysozyme	Label-free	--	6-carboxyfluorescein	Signal-ON	saliva	200 pM	[141]
	Label-free	--	6-carboxyfluorescein	Signal-ON	buffer solution	0.125 µg/mL	[142]
	Label-free	--	Pyrene	Fluorescence emission shift	serum	200 pM	[143]
	Covalent	6-carboxyfluorescein	--	Fluorescence anisotropy increase	saliva	4.9 nM	[144]
	Covalent	6-carboxyfluorescein	--	Signal-ON	buffer solution	0.80 µg/mL	[145]

^a^ Covalently attached to the aptamer; ^b^ Covalently attached to the aptamer or free in solution; ^c^ Covalently attached to non-aptameric oligonucleotide sequences, to the target or free in solution; PDGF = platelet-derived growth factor; VEGF = vascular endothelial growth factor; PTK7 = protein tyrosine kinase 7; AuNP = gold nanoparticle; SWCT = single-walled carbon nanotube; MNP = magnetic nanoparticle; MPA = hydrophilic cyanine dye multiplex probe amplification; NMM = *N*-methylmesoporphyrin IX; TOTO = 1,1′-(4,4,8,8-tetramethyl-4,8-diazaundecamethylene)-bis-4-[3-methyl-2,3-dihydro(benzo-1,3-thiazole)-2-methylidene] quinolinium tetraiodide; n.d. = not determined.
